# Long term expansion profile of mesenchymal stromal cells at protein nanosheet-stabilised bioemulsions for next generation cell culture microcarriers

**DOI:** 10.1016/j.mtbio.2021.100159

**Published:** 2021-11-16

**Authors:** Lihui Peng, Julien E. Gautrot

**Affiliations:** a Institute of Bioengineering and, UK; b School of Engineering and Materials Science, Queen Mary, University of London, Mile End Road, London, E1 4NS, UK

**Keywords:** Mesenchymal stem cell, Emulsion, Protein nanosheet, Microdroplet, Microcarrier, Cell manufacturing

## Abstract

Tremendous progress in the identification, isolation and expansion of stem cells has allowed their application in regenerative medicine and tissue engineering, and their use as advanced in vitro models. As a result, stem cell manufacturing increasingly requires scale up, parallelisation and automation. However, solid substrates currently used for the culture of adherent cells are poorly adapted for such applications, owing to their difficult processing from cell products, relatively high costs and their typical reliance on difficult to recycle plastics and microplastics. In this work, we show that bioemulsions formed of microdroplets stabilised by protein nanosheets displaying strong interfacial mechanics are well-suited for the scale up of adherent stem cells such as mesenchymal stromal cells (MSCs). We demonstrate that, over multiple passages (up to passage 10), MSCs retain comparable phenotypes when cultured on such bioemulsions, solid microcarriers (Synthemax II) and classic 2D tissue culture polystyrene. Phenotyping (cell proliferation, morphometry, flow cytometry and differentiation assays) of MSCs cultured for multiple passages on these systems indicate that, although stemness is lost at late passages when cultured on these different substrates, stem cell phenotypes remained comparable between different culture conditions, at any given passage. Hence our study validates the use of bioemulsions for the long term expansion of adherent stem cells and paves the way to the design of novel 3D bioreactors based on microdroplet microcarriers.

## Graphical abstract

**Figure:**
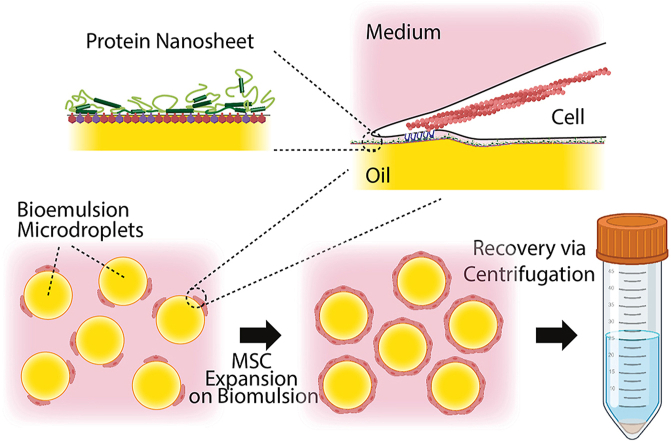

## 1 Introduction

Since the discovery of mesenchymal stromal cells (MSCs) in bone marrow in the late 1960s [1,2], MSCs have been isolated from almost every tissue in the human body [3,4]. MSCs were defined by the International Society for Cellular Therapy (ISCT) as a plastic-adherent cell that maintain self-renewal properties and differentiating potential towards adipogenic, osteogenic and chondrogenic lineages. MSCs should also express CD73, CD90 and CD105 whilst remaining negative for CD45, CD34, CD14 or CD11b, CD79a or CD19 and HLA-DR [5]. Because of their self-renewal ability and multi-potency, they are one of the most widely used cell source for clinical applications, whether for cell therapy or tissue engineering [[5], [6], [7], [8]]. The number of clinical trials registered using MSCs has steadily increased in recent years [2,4], since their first use in a clinical trial in 1995 [9]. On-going clinical explorations of the use of MSCs include treatment for acute graft-versus-host disease (GVHD), bone and cartilage disease, as well as cardiovascular and myocardial infarction repair strategies [2,10]. More recent studies also revealed the potential of MSCs to treat Covid-19 induced pneumonia [11,12]. In most of these applications, high cell numbers need to be delivered, often in the tens of millions per treatment. For example, in the case of GVHD treatment, doses ranging from 0.23 to 9 million cells per kilogram body weight were used per infusion [13,14]. Since MSCs are relatively sparse and rather difficult to isolate from patients in large numbers, novel cell manufacturing platforms are required for the scale up and automation of processes.

To scale up stem cell manufacturing, several types of bioreactors have been developed. Amongst these, bioreactors based on microcarriers, either cultured in conical flasks (similar to bacterial or yeast culture), stirred tanks or expandable bag systems, have been particularly successful [15,16]. Microcarriers, with diameters generally ranging from 100 to 300 ​μm, can be fabricated from a range of biomaterials such as dextran, cellulose, polystyrene and polyvinyl acetate [8,17,18]. Owing to the large surface-to-volume ratio, microcarriers can provide significantly larger surface areas than traditional 2D culture plates and are particularly appropriate for the culture of a broad range of anchorage-dependent cells including MSCs. In addition to their increased volumetric output, microcarrier cultures are also advantageous to allow cell passaging via bead-to-bead transfer without enzymatic treatment [8,19]. This not only saves intensive labour required for cell passaging but also minimises the risk of contamination. The selection of suitable microcarriers is key to the success of cell culture scale up as it can impact the growth kinetics and phenotype of expanded cells [20]. Suitable microcarriers should provide sufficient anchorage for cell growth, allow the expanded cells to be easily and efficiently harvested, and should also display adequate physical mechanical properties to be applied into dynamic systems. The separation of microcarriers from cell products, especially at larger scales (>L) is also an important hurdle to their use. Finally, the cost of microcarriers should be reduced in order to reduce the cost of cell-based therapies and cell products at industrial scales. Currently, the cost of microcarriers for the culture of 1 billion cells is in the range of £800–1900, corresponding to 40–65% of the cost of consumables for cell culture and recovery (based on calculations made for MSC cultures), the media corresponding to most of the rest of this cost [18,21]. Several types of commercial microcarriers have been explored to optimise the culture of MSCs [8,17,18,22,23]. Although quantitative comparison of this data is difficult, those studies have unanimously highlighted the importance of the surface coating of microcarriers to promote cell adhesion, proliferation and the retention of multipotent phenotype. Common ECM protein-coated microcarriers used for MSC culture include cross-linked dextran-based and denatured collagen-coated microporous Cytodex 3 and a cross-linked gelatin-based macroporous CultiSpher-S [18,[23], [24], [25], [26], [27], [28]]. More recently, the adhesion peptide grafted synthetic polymer-coated polystyrene Synthemax® II microcarriers have become one of the most frequently used microcarriers for MSC culture [8,17,28]. For example, Synthemax® II microcarriers allowed the continuous culture of MSCs for more than 50 days (equivalent to 9 passages) with retention of phenotypic marker expression (CD73 and CD105) and trilineage differentiating potential (adipogenesis, osteogenesis and chondrogenesis) [28,29]. However, the long term (multiple passages) benefits of microcarrier-based culture on cell phenotype and the absence of enzymatic treatment and cell re-suspension typically associated with culture on traditional 2D plates have not been systematically quantified.

Overall, important drawbacks that remain in the field of microcarrier design are the difficulty to process cellular products post culture (i.e. separating cells from solid microcarriers), often requiring damaging enzymatic digestion that may impact on cell phenotype and damage to cell membrane receptors [21]. Moreover, enzymatic digestion might not be practical on large scale production as the concentration of enzyme and the incubation times are difficult to control, which would severely impair cell viability and recovery efficiency [21,30]. The high cost of microcarriers also remains a critical issue. Alternative methods avoiding enzymatic treatments include cell culture on thermoresponsive microcarriers (e.g. based on poly(N-isopropyl acrylacmide), PNIPAAm), from which cells and cell colonies can spontaneously detach by simple decrease of the temperature below the lower critical solution temperature (LCST) of PNIPAAm [31,32]. However, this often leads to incomplete detachment and still requires separation of suspended cells from solid microcarriers [25]. Other approaches include the culture of cells on dissolvable microcarriers which can be easily removed by non-invasive treatments such as pectinase digestion [21,33], though the microcarriers are now discontinued [34]. Overall, in all strategies, additional processing steps and high costs remain important hurdles to the adoption of these platforms for the scale up of cell manufacturing. Microplastics are also increasingly becoming unappealing from an environmental point of view and their potential contamination of biological products may pause regulatory hurdles to their long term clinical implementation.

In the last decade, microdroplet microfluidic and flow through technologies have revolutionised the field of single cell sequencing, high throughput monitoring and screening [35,36]. In addition, biphasic manufacturing platforms such as emulsions are particularly attractive for the synthesis and purification (e.g. via extraction) of fine chemicals, therapeutic molecules, polymers and nanomaterials, and are widely applied in chemical engineering and manufacturing. However, the implementation of microdroplet platforms for adherent cell culture and stem cell technologies has remained limited, as thought to be incompatible with the importance of substrate mechanics in regulating cell adhesion and stem cell phenotype [37,38].

Cell adhesion to biomaterials, mediated by integrin binding to extra-cellular matrix proteins and ligands, is indeed regulated by matrix mechancis [[39], [40], [41]]. In turn, this process regulates the actomyosin cytoskeleton assembly and contractility and a wide range of phenotypes, via mechanisms such as ERK, SMAD, MAL/SRF and YAP signalling [40,42,43]. At first glance, cell adhesion to liquids (e.g. oils) is therefore unexpected. However, in the 1980s, Keese and Giaever reported that some liquids enabled the adhesion and proliferation of fibroblasts and that this phenomenon required supplementation with surfactant molecules [44,45]. The precise mechanism enabling this process remained unclear. More recently we reported that cell adhesion to low viscosity liquids, including fluorinated and silicone oils was dependant on the self-assembly of strong protein nanosheets at corresponding liquid-liquid interfaces [[46], [47], [48]]. We demonstrated that co-surfactant molecules such as pentafluorobenzoyl chloride (PFBC) modulate the interfacial mechanics of protein nanosheets, in turn regulating cell adhesion via the classic integrin-mediated actomyosin machinery [47,48]. This allowed the culture of a broad range of cell types, including human primary keratinocytes and MSCs [[46], [47], [48]], fibroblasts [44,45,49,50] and myoblasts [51,52]. In some cases, ECM proteins were found to directly assemble at the surface of some liquids and promote cell adhesion and culture on 2D liquid interfaces [53], but this remains difficult to control and does not support the stabilisation of associated emulsions for application in 3D bioreactors. Therefore protein nanosheet technologies appear attractive to mediate ECM protein and ligand functionalisation whilst controlling interfacial mechanics and emulsion stability. Although the proof of concept of these systems and the simplification of cell processing (e.g. via centrifugation) have been demonstrated, the long term expansion of stem cells on bioemulsions and the characterisation of their phenotype has not been established yet.

**Fig. 1:**
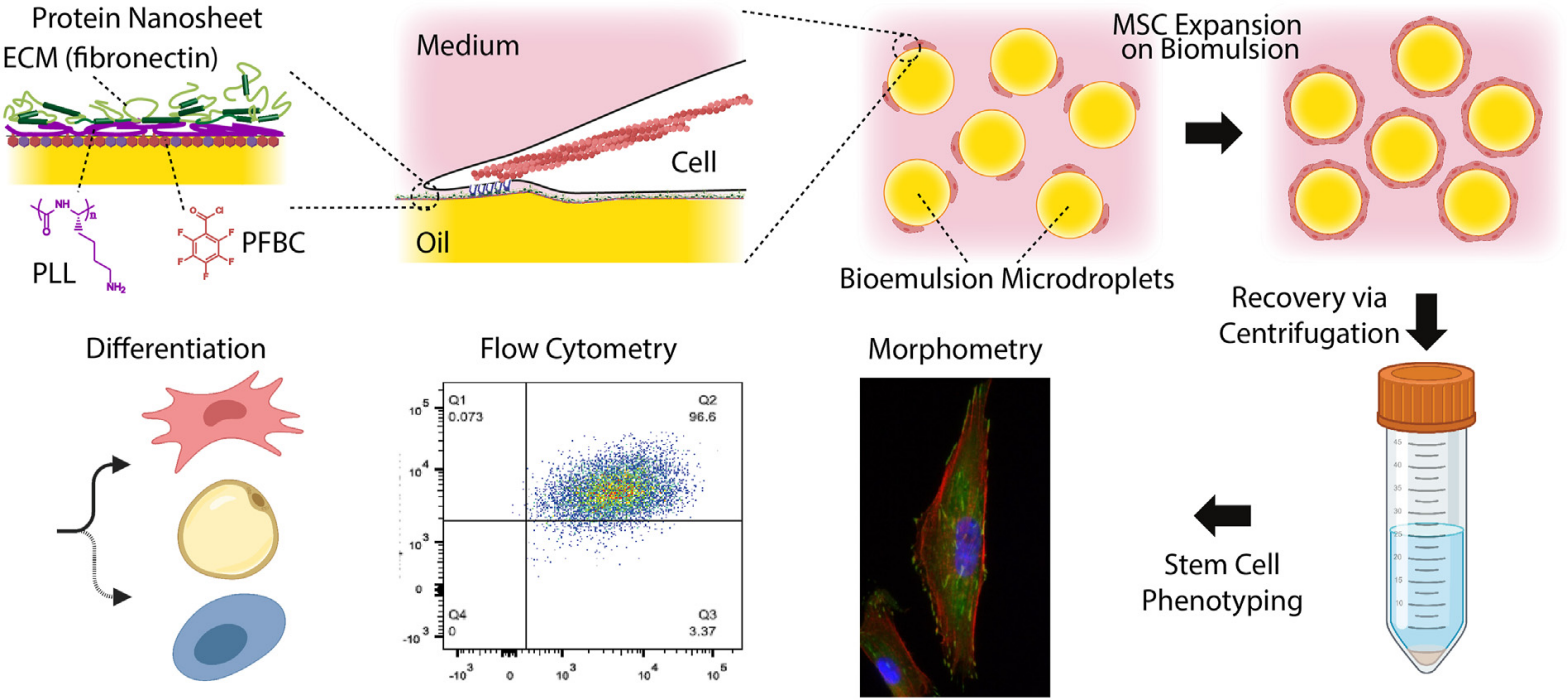
Protein nanosheets self-assembled at liquid-liquid interfaces allow the stabilisation of bioemulsions. Their strong interfacial mechanical properties allow the resulting interfaces to resist cell-mediated contractile forces and regulate cell spreading. Following expansion for multiple passages, cells are harvested (via centrifugation) and their stem cell phenotype is characterised via morphometry, flow cytometry and cell differentiation assays.

In this work, we generate bioemulsions stabilised by poly(L-lysine) nanosheets, co-assembled with the surfactant PFBC and systematically characterise the long term expansion of MSCs at their surface. These nanosheets were selected based on a previous study in which we reported the ability to support cell adhesion to liquid interfaces and studied some of the parameters regulating their self-assembly [48]. We present the first side-by-side comparison of the performance of bioemulsion microcarriers with solid microcarriers (commercial Synthemax® II) and 2D tissue culture flasks for the long term (multiple passages) expansion of MSCs (Fig. 1). We characterise the morphology of cells produced at different passages, their overall expansion and expression of stem cell markers via PCR and flow cytometry. We characterise the retention of multi-potent phenotypes after different culture periods by inducing differentiation into osteo-, adipo- and chondrogenic lineages and characterising the phenotype of resulting cells. Overall, our results demonstrate that MSC phenotypes after long term expansion on bioemulsions, solid microcarriers and 2D culture flasks are comparable. We observe that the stage of cell expansion (passage number) has a far more important impact on MSC phenotype than any difference between the 3 culture platforms studied, at any given time. Therefore, our study demonstrates the feasibility of protein nanosheet-stabilised bioemulsions for the long term expansion of stem cells and their application in 3D bioreactors for the production of stem cells with preserved phenotype.

## 2 Materials and methods

### 2.1 Generation of protein nanosheet-stabilised bioemulsions

1 ​mL fluorinated oil (Novec 7500, ACOTA) containing the fluorinated surfactant 2,3,4,5,6-Pentafluorobenzoyl chloride (PFBC, Sigma-Aldrich) at a final concentration of 10 ​μg/mL and 2 ​mL of poly(L-lysine) (PLL) solution (200 ​μg/mL, Sigma-Aldrich) in PBS (pH adjusted to 10.5) were added in a 15 ​mL centrifuge tube. The tube was vigorously shaken via vortexing for 15 ​s, to mix both phases and generate the emulsion and subsequently left to incubate at room temperature for 1 ​h. The top liquid phase, above the settled emulsion was aspirated and replaced with PBS 6 times. Human plasma fibronectin (FN, Sigma-Aldrich) was deposited at the surface of oil droplets after PLL adsorption. 20 ​μL of FN (1 ​mg/mL) was added (final concentration of 10 ​μg/mL) and incubated at room temperature for 1 ​h. The top liquid phase above the bioemulsion was aspirated and replaced with PBS 6 times. For cell seeding, 500 ​μL of growth medium were added in a 24 well plate treated with 300 ​μL poly(L-lysine)-*graft*-polyethylene glycol (PLL-g-PEG, SuSoS AG, 25 ​μg/mL) for an hour and washed with PBS three times. 120 ​μL of the bioemulsion was then transferred to each well before 500 ​μL of growth medium containing MSCs at the desired density was added.

To characterise the bioemulsion surface area, bioemulsions were imaged via bright field microscopy. Images were analysed by outlining microdroplets via imageJ using the Trainable Weka Segmentation plugin, allowing the measurement of their diameter and surface area. For fluorescence imaging, Alex Fluor 594-conjugated PLL was used. The conjugated PLL stock solution was prepared by dissolving PLL powder in 0.1 ​M NaHCO_3_ buffer and reacting with Alexa Fluor 594 NHS ester (succinimidyl ester, Sigma-Aldrich) dissolved in DMSO. The conjugated PLL was mixed with non-labelled PLL at 1:9 ratio and added into each well to a 100 ​μg/mL final concentration. The incubation and washing process was the same as the one described above.

### 2.2 Mesenchymal stromal cell (MSC) culture, cryopreservation and thawing

Bone marrow derived human MSCs were obtained from PromoCell and cultured from passage 2 on T75 flasks in MSC growth medium (PromoCell) in an incubator (37 ​°C and 5% CO_2_). Medium changed every other day. When 70–80% confluency was reached, MSCs were harvested with 4 ​mL Accutase solution (PromoCell), centrifuged, counted and resuspended in freezing medium (Cryo-SFM, PromoCell) at the 500,000/mL. 1 ​mL of cell suspension was transferred to each cryovial and stored in a Mr. Frosty™ freezing container. The container was kept at −80 ​°C for 24 ​h before transferring the vials to a cell bank filled with liquid nitrogen. The same batch of cells was not cryopreserved more than once. For long-term culture experiments, MSCs were thawed at passage 3 on T75 flasks in the growth medium. When the confluency was reached, the cells were harvested for real-time PCR (RT-PCR), flow cytometry, as well as seeded to the corresponding substrates at 20,000 ​cells per well to start long-term expansion experiments.

### 2.3 Long-term MSC culture, passaging and harvesting on microcarriers

To avoid cell adhesion to tissue culture plastic (TPS) and restrict cell adhesion to bioemulsions or solid microcarriers, 300 ​μL of 25 ​μg/mL PLL-g-PEG solution were used to coat each well of a 24-well plate and left to incubate for 1 ​h. The PLL-g-PEG solutions were then aspirated and wells were washed with PBS twice before 500 ​μL growth medium was added into each well. 120 ​μL of bioemulsion (or 20 ​mg of Synthemax® II microcarriers pre-incubated with 500 ​μL MSC growth medium) were then transferred to each well, allowing the microcarrier suspension to fully cover of the surface of the well. MSCs were then gently seeded at a density of 20,000 ​cells/well and placed in an incubator without shaking for 24 ​h. Hence the densities of MSCs seeded per cell adhesive area were 2,100, 4200 and 2800 ​cells/cm^2^, for TPS, Synthemax® II and bioemulsions, respectively. We note that such rest period, or alternative methodologies, could be optimised to improve cell seeding efficiency, but this was not further studied in this work. The well-plate was then placed on an orbital shaker (VWR) and agitated at a speed of 70 ​rpm, in an incubator (37 ​°C and 5% CO_2_). When 70–80% cell confluence was reached, cell passaging on emulsions and mirocarriers was carried out by transferring 1/3 of the bioemulsion/microcarrier suspension to a new well (coated with PLL-g-PEG) containing fresh bioemulsion/microcarriers with medium. This allowed cell migration from droplet-to-droplet (or microcarrier-to-microcarrier) without requiring the use of enzymatic treatment and cell detachment. The well-plate was placed on an orbital shaker (VWR, Stuart™ Gyratory rocker, SSL3) and agitated at a speed of 40 ​rpm, in an incubator (37 ​°C and 5% CO_2_). After 24 ​h, the speed was increased to 70 ​rpm.

To harvest cells from bioemulsions, the bioemulsion was transferred by pipetting to a centrifuge tube, the residual excess medium was removed before the emulsion was washed with PBS three times. The tube was then centrifuged for 5 ​min at a speed of 1500 ​rpm to break the bioemulsion. The cell pellets were collected at the interface between the PBS and oil phases. For cells cultured on microcarriers, the microcarriers were transferred in a centrifuge tube by pipetting. When the microcarriers were settled at the bottom of the tube, the medium was removed before the PBS was added into the tube to wash off excess medium. The microcarriers were incubated with Accutase solution for 5 ​min in an incubator for cell detachment. The solution was transferred to another centrifuge tube capped with a cell strainer to separate detached cells from the microcarriers. The flow through was saved and the cell pellet was collected after 5 ​min centrifugation at a speed of 1200 ​rpm.

### 2.4 DNA quantification for characterisation of cell proliferation

Cell densities were determined via DNA quantification using CyQUANT™. Cells cultured from corresponding substrates (TPS/bioemulsion/microcarriers) were collected at desired time points (as indicated above), transferred into a 2 ​mL microcentrifuge tube and washed with PBS three times before storage at −70 ​°C, to induce cell lysis. Resulting suspensions were thawed at room temperature and incubated with CyQUANT™ kit solutions, following protocols outlined by the manufacturer. The resulting solution was then transferred into a 96-well plate and the fluorescence intensity was quantified using a fluorescence microplate reader set up with excitation at 480 ​nm and emission at 520 ​nm. To convert absorbances into cell densities, standard curves generated from known cell densities (but recovered from TPS and bioemulsion/microcarriers using identical protocols to those used for each system; to account for the impact different substrate may have on recovery and DNA quantification, three separate calibration curves were generated) were generated (see Supplementary Fig. S1). The population doubling time was calculated as (t_2_-t_1_)/(3.32∗(log(N_2_)-Iog(N_1_))), where t_1_ and t_2_ are the start and end time points of the experiments, respectively, and N_1_ and N_2_ are the cell densities at the start and end time points, respectively, measured via the CyQUANT™ assay.

### 2.5 Immunostaining and fluorescence imaging

Cell adhesion was studied via immunostaining of adherent cells (vinculin and phalloidin). Cells were harvested from different substrates as described above and reseeded (5000 ​cells/well, 500 ​μL MSC expansion medium) on glass coverslips pre-coated with FN in 24-well plates. After 24 ​h, the samples were washed with PBS and fixed with 500 ​μL 4% PFA for 10 ​min, washed with PBS, before permeabilization with 500 ​μL 0.4% Triton X-100 solution (in PBS) for 5 ​min at room temperature. This was followed by 1 ​h blocking (PBS containing 3 ​wt% BSA), with simultaneous staining for actin by introducing tetramethyl rhodamine isothiocyanate phalloidin in the bocking solution (Sigma-Aldrich, 1:1000). Samples were subsequently incubated with the corresponding primary antibody (Sigma-Aldrich, mouse anti-vinculin, 1:400 in blocking buffer) for 1 ​h at room temperature, washed with PBS and then incubated with Alexa Fluor 488-conjugated secondary antibody (Sigma-Aldrich, goat-anti-mouse 1:1000 in blocking buffer) and Dapi (Sigma-Aldrich, 1:1000 in blocking buffer) for 1 ​h at room temperature. After washing in PBS and deionised water, stained samples were mounted in Mowiol on coverslips and imaged using a Leica DMI4000B epifluorescence microscope. To quantify cell spreading and morphology, cytoskeleton images were analysed using thresholding and watershedding protocols in ImageJ. Focal adhesions were analysed by ImageJ using a previously reported protocol.^59^ Focal adhesion sized between 0.5 and 10 ​μm^2^ were considered for this analysis.

### 2.6 Live imaging

Live imaging of cells migrating from one microdroplet to another or from droplets to solid stubstrates was performed to examine their ability to be directly transferred without enzymatic digestion. For droplet to droplet transfer, bioemulsions at the surface of which confluent cells (MSCs) had been allowed to grow were transferred to a new well containing freshly prepared microdroplets (PLL and fibronectin coated as above), in growth medium. For droplet to solid transfer, bioemulsions at the surface of which confluent cells (MSCs) had been allowed to grow were transferred to a new well (non-coated with PLL-g-PEG) in growth medium. A Lumascope 720 microscope was used to image wells every 15 ​min over a period of 20 ​h (the video is run at 15 frames per second).

### 2.7 MSC differentiation and phenotypic characterization

MSCs were cultured at a density of 50,000 ​cells per well in 48-well plates. After 24–72 ​h incubation, when 100% confluency was reached, differentiation was induced by replacing the growth medium with either osteogenic differentiation or adipogenic differentiation medium (PromoCell), whilst control groups were kept in culture in growth medium. The media were replaced every three-days over a period of two weeks. For chondrogenesis, cells were seeded in U-bottom 96-well plates (Greiner Bio-One) for 72 ​h to allow the formation of spheroids. Chondrogenesis was induced by culturing in chondrogenic differentiation medium (PromoCell) while the undifferentiated control group was cultured with Dulbecco's Modified Eagle's Medium (DMEM, low glucose, Sigma-Aldrich) with 2 ​mM L-glutamine and 10% FBS (PAA). The media were replaced three times a week for a period of three weeks.

#### 2.7.1 Oil red staining

Oil Red solutions allow to stain (red-orange) lipid droplets produced during adipogenesis. Oil Red staining stock solutions were prepared by dissolving 150 ​mg of Oil Red O powder (Sigma-Aldrich) with 50 ​mL of 99% isopropanol. The solution was filtered and stored in the dark after the powder was fully dissolved. The working solution was prepared by mixing the Oil Red stock solution with DI water at 3:2 ratio for 10 ​min, the mixture was then filtered via a 0.2 ​μm filter. Samples were washed twice with PBS before being fixed with 4% PFA for 10 ​min. After which, the samples were washed three times with PBS to remove excess PFA and then stained with the Oil Red working solutions. After incubation for 1 ​h, samples were washed six times with PBS and imaged by bright field microscopy.

#### 2.7.2 Alkaline phosphatase (ALP) staining

Solutions were prepared by dissolving one 5-bromo-4-chloro-3-indolyl phosphate/nitro blue tetrazolium tablet (BCIP/NBT, Sigma-Aldrich) in 10 ​mL DI water. Samples were washed with 0.1 ​M PBS three times and then fixed in 90% ice cold ethanol for 4 ​min. After washing three times with DI water, BCIP/NBT working solutions were added to each sample and incubated for 1 ​h at room temperature. After incubation, samples were washed three times with DI water and imaged by bright field microscopy.

#### 2.7.3 Alizarin Red staining

Alizarin Red solutions were prepared by dissolving 1 ​g Alizarin Red S powder (Sigma-Aldrich) in 50 ​mL DI water. After the pH of the solution was adjusted just below 4.2 with hydrochloric acid, the solution was filtered using a 0.2 ​μm filter and stored in the dark. Samples were washed twice with PBS and fixed with 4% PFA for 10 ​min. Samples were washed three times with DI water, then incubated with the Alizarin Red working solution. After 1 ​h incubation, samples were washed six times with DI water and imaged by bright field microscopy.

#### 2.7.4 Alcian Blue staining

Working Alcian Blue staining solutions were prepared by dissolving 10 ​mg Alcian Blue 8 GX (Sigma-Aldrich) in solutions of 6 ​mL ethanol and 4 ​mL acetic acid. A de-staining washing solution was prepared by mixing 12 ​mL ethanol with 8 ​mL acetic acid. After washing twice with PBS to remove excess medium, the spheroid was fixed with 4% PFA for 45 ​min at room temperature. After rinsing with PBS three times, the sample was left in the Alcian Blue working staining solution overnight. The staining solution was carefully removed and the spheroid was washed three times with the destaining solution for 10 ​min, prior to taking images with a camera.

### 2.8 Flow cytometry

For flow cytometry, the antibody panels were designed as CD105-FITC, CD73-RPE and CD-90-APC or CD105-RPE, CD73-FITC and CD90-APC for positive panels, CD34-FITC, CD45-RPE and CD19-Pacific Blue for negative panels (Bio-Rad). Unstained controls, single colour controls and fluorescence minus one control (FMO, samples are stained for all the flurochromes in the panel, except for one) were also performed respectively for voltage adjustment, compensation and positive gating. All the antibodies were prepared in PBS containing 1% BSA at dilutions suggested by the supplier. After culture on TPS, bioemulsions or solid microcarriers at passage 4, 6, 8 and 10 (from cells at passage 3), MSCs were harvested by accutase solution treatment, as single cell suspensions, from the corresponding substrates and transferred to 2 ​mL microcentrifuge tubes at a density of 1 ​× ​10^6^ ​cells/tube. Cells from each substrate were individually stained with one of the panels, at a volume of 100 ​μL for 30 ​min on ice. After washing with 1.5 ​mL PBS, each microcentrifuge tube was filled with 300 ​μL ice cold PBS. Flow cytometry was performed on a FACS Canto II instrument (BD Biosciences). 405 Coherent VioFlame, 488 Coherent Sapphire and 635 JDS Uniphase HeNe lasers were used for excitation and sorting. The results were analysed by Flowjo. For data analysis, gating was performed to discard dead cells, debris and cell doublets before CD73 positive cells were selected on histograms first (CD73 is highly expressed thus can be easily discriminated from negative signals in histograms), based on the FMO control. Cells co-expressing CD105 and CD90 were then sub-gated from the dot plot, based on the corresponding FMO control.

### 2.9 Real-time polymerase chain reaction (RT-PCR)

The RNA of MSCs (harvested after culture at the desired time point, on the substrate of interest) was extracted via a Qiagen RNeasy Kit according to the protocol from the supplier. The concentration of extracted RNA was measured via Nanodrop (Thermo Fisher) and the RNA was then reverse-transcribed using QuantiTect® Reverse Transcription Kit (Qiagen) following the protocol from the supplier. The cDNA obtained was preserved at −20 ​°C. RT-PCR experiments were set up and performed following the Taqman® Gene Expression Assay protocol using the kit in a QuantStudio™ Real-Time PCR System (Applied Biosystems™). Beta-2-microglobulin (B2M, Hs00187842_m1, Thermo Fisher) was selected as the housekeeping gene, and results were shown as the relative value obtained, comparing all samples to newly thawed P3 cells controls. Since some of the differentiation markers did not express under growth medium condition, the P4 cells cultured on TPS in differentiation medium were used as controls for differentiation. Analysis of the results was performed according to 2^−ΔΔCt^ method. Details of target genes are presented in the Supplementary Table S1.

### 2.10 Statistical analysis

Statistical analysis was carried out using Origin 2019 through one-way ANOVA with Tukey test for posthoc analysis. Significance was determined as ∗ P ​< ​0.05, ∗∗P ​< ​0.01, ∗∗∗P ​< ​0.001 and n.s., non-significant. Throughout the manuscript, SD abbreviates standard deviation. S.E. abbreviates standard error.

## 3 Results and discussion

The selection of protein nanosheets for the stabilisation of bioemulsions supporting MSC adhesion and expansion was based on several key criteria: 1. The formation of mechanically strong protein nanosheets able to resist cell-mediated contractile forces generated during cell spreading and migration; 2. The ability to readily adsorb ECM proteins at the surface of resulting protein nanosheets; 3. The stabilisation of oil microdroplets resulting in bioemulsions that persist during cell culture. We previously identified two types of protein nanosheets displaying strong interfacial mechanics and supporting cell adhesion and expansion [47,48]. Albumin nanosheets assemble fast in physiological conditions, with ultimate interfacial moduli in the range of 10–40 ​mN/m [47]. Poly(L-lysine) (PLL) nanosheets form stiffer interfaces (interfacial moduli near 3 ​N/m), but also enable rapid fibronectin adsorption owing to their positive charge [48]. Although cell adhesion to liquids has also been reported through simple adsorption from medium proteins (often introduced via the serum used) and direct fibronectin adsorption, we did not select these systems as we found that the former was not supporting MSC adhesion (especially on emulsions displaying high surface curvature), whereas the latter did not promote the formation of stable emulsions and was reported to be very dependent on oil type. However, the stability of PLL nanosheet stabilised bioemulsions in culture conditions had not been investigated. The fluorinated oil Novec 7500 was selected owing to its high density, cytocompatibility and use in biotechnology applications and microdroplet microfluidic systems [54].

**Fig. 2:**
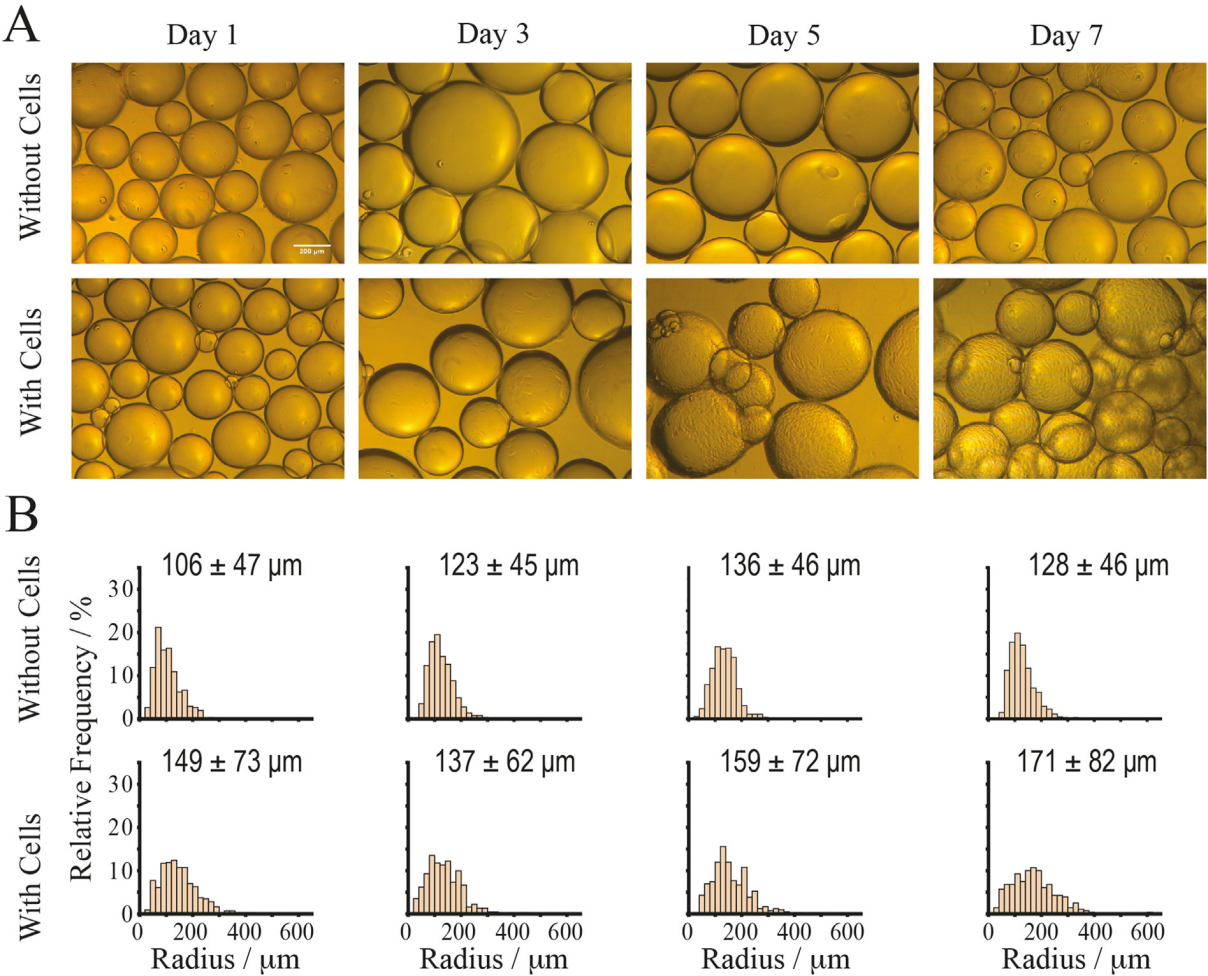
The stability of PLL/FN bioemulsions is maintained in the presence of MSCs over a period of 7 days. A) Bright field microscopy images of bioemulsions with and without MSCs over a period of one week (under agitation) and B) corresponding histograms of size distributions and average radii ​± ​SD.

Therefore, we first investigated the long-term stability of PLL-stabilised bioemulsions in culture conditions, including the impact of high cell densities on microdroplet stability over a period of 7 days. Over this time period, we observed no apparent flocculation of emulsions or phase separation. Emulsions maintained a spherical shape without significant distortion (Fig. 2A). This was confirmed by analysing the size distribution of emulsions in the absence and presence of cells (Fig. 2B). Histograms indicate a slight broadening of the emulsion size distribution, although the average diameter only increased from 149 to 171 ​μm. The average volume of droplets was found to increase after cell seeding and increased slightly at later time points (Supplementary Fig. S2), but without macroscopic phase separation of the bioemulsions, whether with or without cell seeding. This could be associated to some level to the destabilisation of some of the droplets in the system, but also perhaps with a slight deformation of the droplets after 5–7 days of culture (perhaps as a result of cell-mediated forces).

**Fig. 3:**
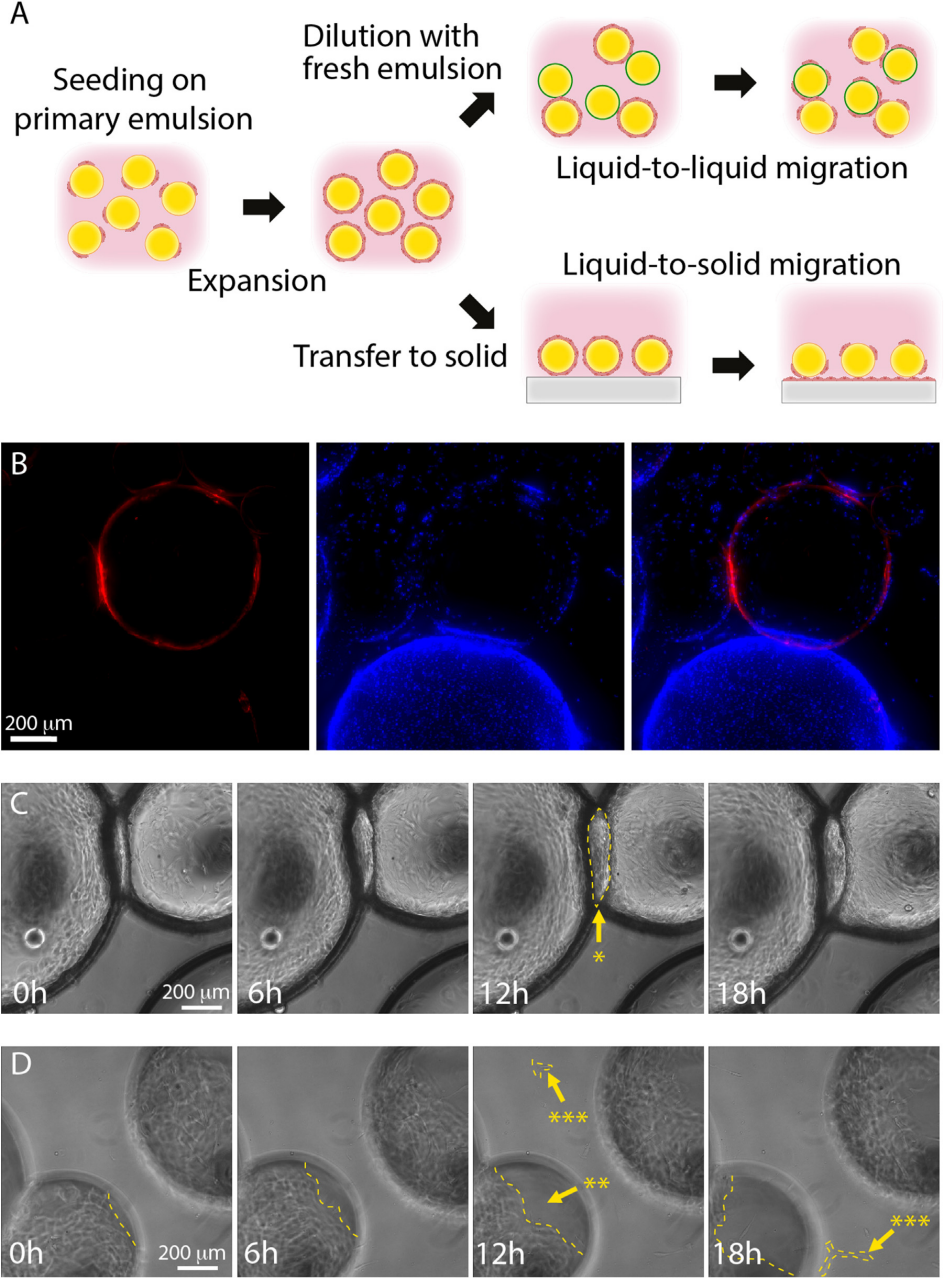
MSCs can be directly transferred from microdroplets, without enzymatic treatment. A) Schematic representation of direct transfer of cells from droplets to other droplets or to solid substrates. B) Epifluorescence microscopy image of MSCs migrating from a confluent droplet to a recently prepared tagged (Alexa Fluor 594-PLL) microdroplet (day 3 after introduction). Blue, nuclei; Red, PLL. C and D) Imaging of droplet to droplet (C) and droplet to solid (D) cell transfer (taken from Supplementary Videos S1 and S2, respectively) indicating the migration of MSCs from microdroplets, during a time course of 18 ​h. Note the bulge developing at the interface between two droplets (indicated by the arrow with ∗), the disappearance of cells from one of the droplets (indicated by the arrow with ∗∗) and the appearance of cells spreading on the solid substrate (indicated by the arrow with ∗∗∗), underlying cell transfer. Dashed lines indicate examples of the cell contours indicated by the arrows.

To investigate whether cells are able to migrate from one bioemulsion droplet to another, thus enabling to bypass cell detachment for passaging, we introduced freshly prepared bioemulsions generated with tagged PLL (Alexa Fluor 594) together with bioemulsions on which MSCs had been cultured for 7 days and reached confluency. 3 days following the introduction of this new bioemulsion, MSCs could be seen to migrate to the new carriers and to populate these new surfaces (Fig. 3). This process was also confirmed via live imaging over a period of 5 ​h (Supplementary Video S1), during which cells bridged the gap between droplets and gradually migrated to fresh carriers. Similarly, MSCs were found to migrate from microdroplets to the underlying substrate, following droplet transfer to cell-adherent wells (Fig. 3D and Supplementary Video S2). We note that other methodologies could be developed to promote faster migration/adhesion to new microcarriers, such as cell collection after centrifugation/detachment and reseeding. We also note that quantification of the number of microcarriers covered by cell colonies may allow identifying some of the factors limiting expansion on microcarriers and would be an aspect deserving attention. However, this step was not further optimised in our study.

Supplementary data related to this article can be found at https://doi.org/10.1016/j.mtbio.2021.100159.

The following are the supplementary data related to this article:

Multimedia component 2 Multimedia component 2

Multimedia component 3 Multimedia component 3

**Fig. 4:**
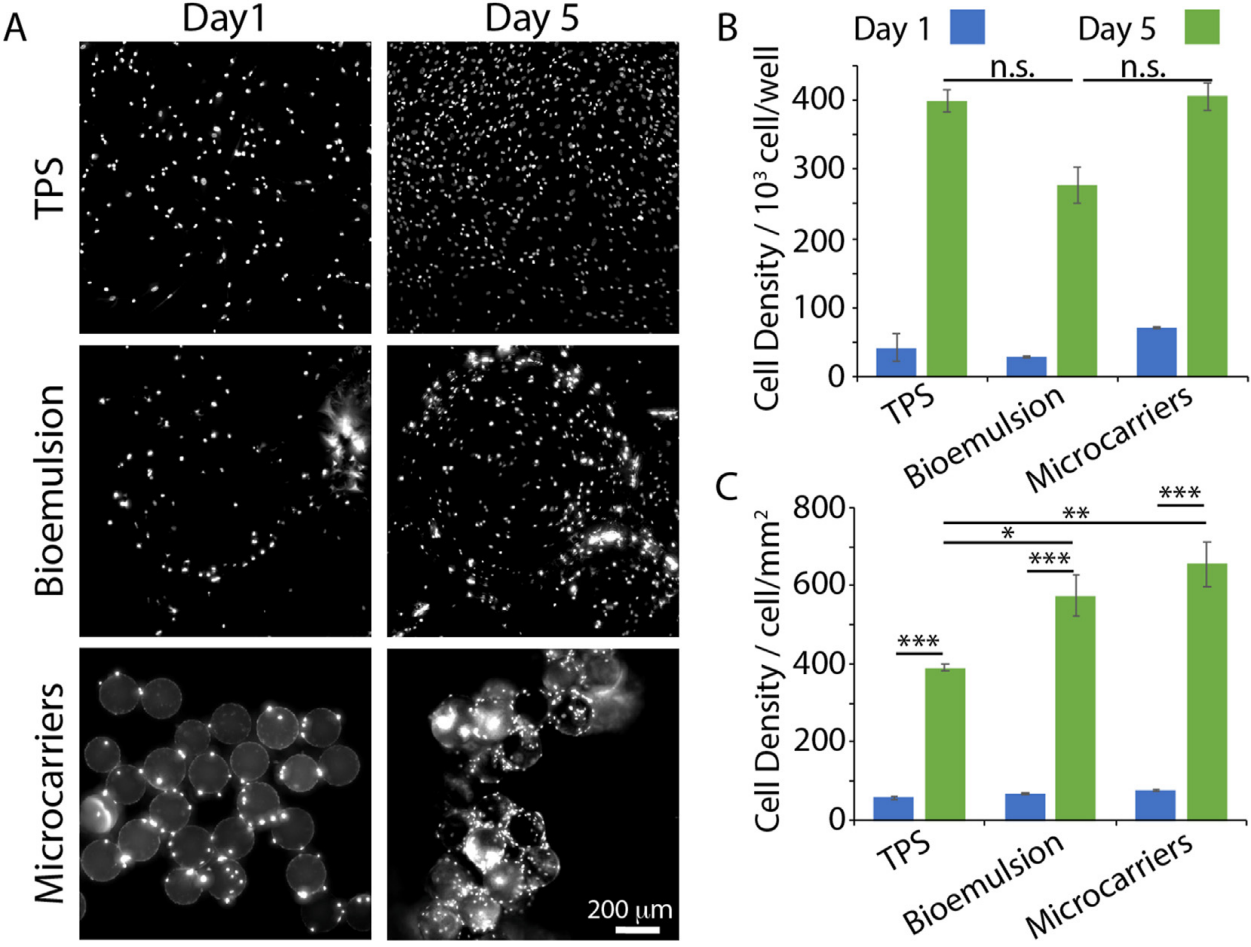
MSC proliferation on TPS, bioemulsions and microcarriers at passage 4. A) Epifluorescence microscopy images of Hoechst stained MSCs cultured on TPS, bioemulsions and microcarriers at days 1 and 5 of the first passage cycle (from passage 3 ​cells). B and C) Corresponding MSC densities quantified via CYQUANT™ assay. Error bars are S.E.; n ​≥ ​3.

For long term expansion studies (equivalent to 6 cycles of expansion/passaging), we compared MSCs cultured on bioemulsions to cultures on the solid microcarriers Synthemax® II as well as conventional culture on 2D tissue culture polystyrene (TPS, 6 well plate). The volumes of bioemulsions and weight of microcarriers introduced in each well of a 24 well plate (with surface passivation with PLL-PEG to prevent cell adhesion to the plastic) corresponded to surface areas of 4.8 and 7.2 ​cm^2^ (compared to 9.6 ​cm^2^ for the surface of a well in a 6-well plate for TPS), respectively, with cell densities per well (20,000 ​cells) and volume of medium (1.5 ​mL) kept constant in all three conditions. Cell densities at different time points and passage times were evaluated via DNA quantification assay (CyQUANT™ assay, Fig. 4; data compared to our standard, Supplementary Fig. S1). At early time points (day 1), we found no difference in cell densities in the different culture systems, suggesting comparable levels of seeding efficiencies (although some impact of cycling cannot be ruled out). After 5 days of culture, cells proliferated in all conditions and were found to cover the surface of microcarriers. Cell densities were found to be comparable in microcarriers (solid or liquid) and were slightly higher than those observed on TPS (p<0.05). Similar trends were observed in subsequent passages (Supplementary Fig. S3). We do note a slight increase in the population doubling time observed on bioemulsions at P6 and P8. However, at passage 10, there was an apparent decrease in cell cycling observed on all substrates, with overall reduced cell densities, consistent with a reduction in cell proliferation reported in the literature [[55], [56], [57]]. This was particularly pronounced in the case of cells cultured on solid microcarriers, for which we measured a particularly strong increase in passage doubling time (Supplementary Fig. S4). Therefore, our results indicate the excellent proliferation potential of MSCs cultured on bioemulsions (comparable to that observed on solid microcarriers) and the overall cell cycling performance that can be achieved on bioemulsions in 3D platforms.

**Fig. 5:**
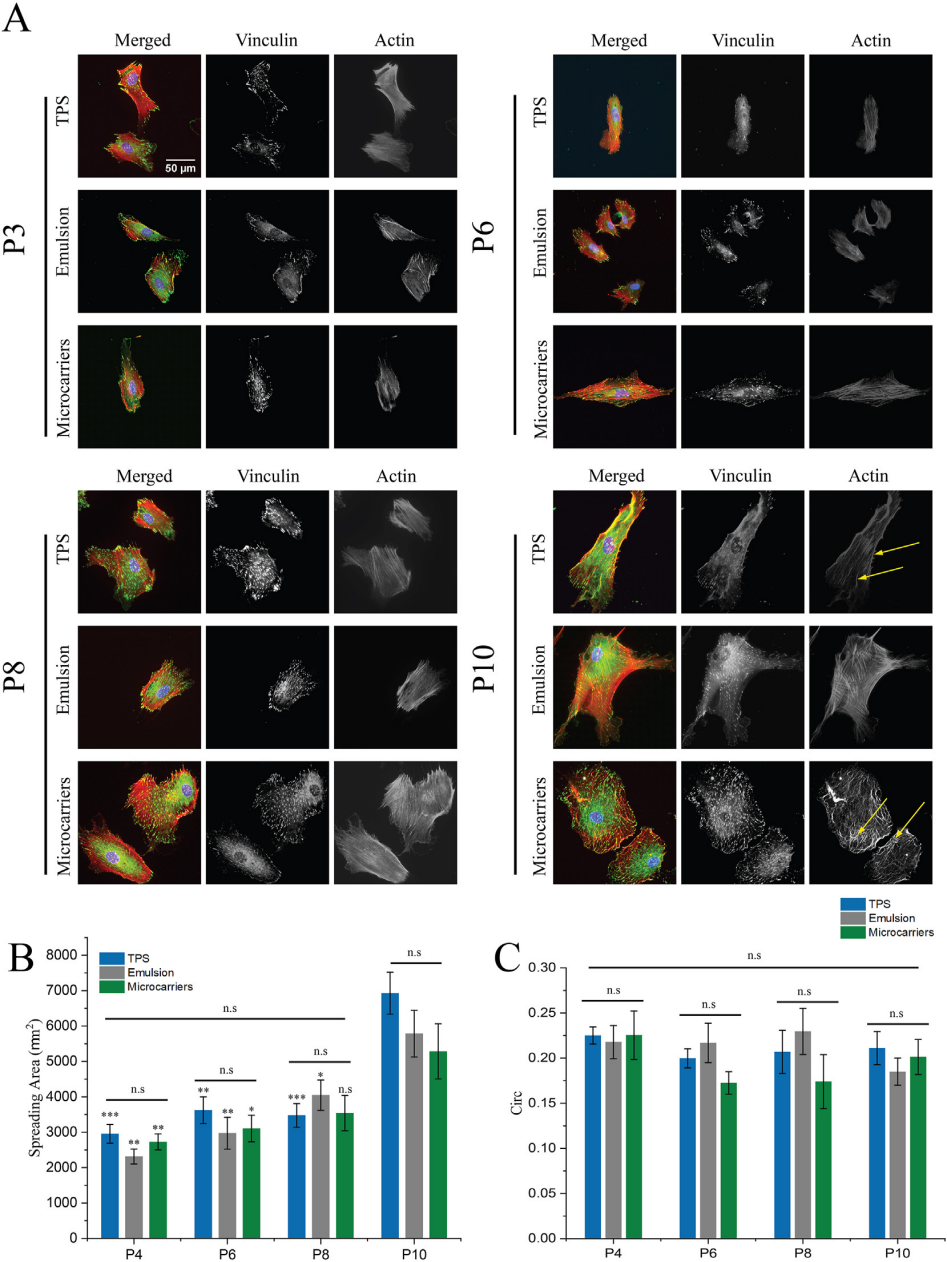
Evolution of MSC spreading and morphology after culture on TPS, bioemulsions and solid microcarriers for 6 passages. A) Confocal microscopy images of MSCs spreading on FN-coated glass slides for 24 ​h, following long term culture on corresponding substrates (green, vinculin; red, actin; blue, DAPI). Arrows indicate examples of poorly organise stress fibres at late passages for cells culture on TPS and microcarriers. Quantification of cell spreading area (B, unless indicated by a bar, statistical comparisons refer to the same condition at P10) and circularity (C, “Circ”), calculated from the analysis of actin images. Error bars are S.E.; n ​≥ ​3, more than 40 ​cells for each condition were measured in each experiment. ∗, p ​< ​0.05; ∗∗, p ​< ​0.01; ∗∗∗, p ​< ​0.001 and n.s., non-significant.

The phenotype of MSCs cultured over prolonged passage times was next examined. Cell spreading and morphology is considered as one important hallmark of the MSC phenotype, and differentiation has been associated with changes in focal adhesion formation and actin reorganisation [58]. Therefore, we quantified the morphology of MSCs cultured at different passages on the three substrates studied. To do so, as cells respond to the biochemistry and mechanics of their microenvironment, MSCs were reseeded on identical fibronectin coated glass substrates allowing direct comparison of morphologies. Cell spreading gradually increased with passage number (in particular at passage 10, Fig. 5), possibly indicating a shift towards osteogenic differentiation or cell senescence [59,60]. No significant difference was observed between cell spreading or morphology for MSCs cultured on the different substrates studied, throughout the culture period when comparisons were made at one specific time point. However, at passage 10, cell spreading areas were almost twice the size of that of cells at passage 4 on all substrates (Fig. 5B). At this late passage, folds in the actin structure indicative of membrane ruffles [61,62] were observed at the periphery of cells that had been cultured on TPS and solid microcarriers. Actin stress fibres were also poorly organised (Fig. 5A). Membrane ruffling is considered as an indicator of inefficient lamellipodia adhesion and enhanced motility [[61], [62], [63]]. Therefore, these observations may suggest a reduction in stable cell adhesion and changes in cytoskeleton assembly, which are also regarded as characteristics of senescent cells [60]. In the case of cell circularity, no significant changes were observed at all time points, on all substrates and MSCs maintained an elongated shape with circularities near 0.23 (Fig. 5C).

To confirm observations made on cell spreading and morphologies, the formation of matrix adhesion was investigated. The number of focal adhesions (quantified from vinculin staining and microscopy), their area, circularity, aspect ratio and size distribution were systematically quantified (Supplementary Fig. S5). No significant difference was observed for any of these parameters when comparing across different substrates or time points (apart from the number of focal adhesions that was overall increased at P10 compared to cells at P4). The increased number of adhesions observed at late time points is in agreement with the overall increased spreading area. The histograms of adhesion size distribution (Supplementary Fig. S5E) revealed that the majority of adhesions were typically smaller than 2 ​μm^2^ ​at early time points, but that the percentage of adhesions below 1 ​μm^2^ increased from P4 (near 33%) to P10 (over 35%). Adhesion areas below 1 ​μm^2^ are typically identified as focal complexes rather than focal adhesions [64,65]. Increased level of focal complexes may therefore reflect increased cell spreading as well as a potential increase in mobility at P10.

**Fig. 6:**
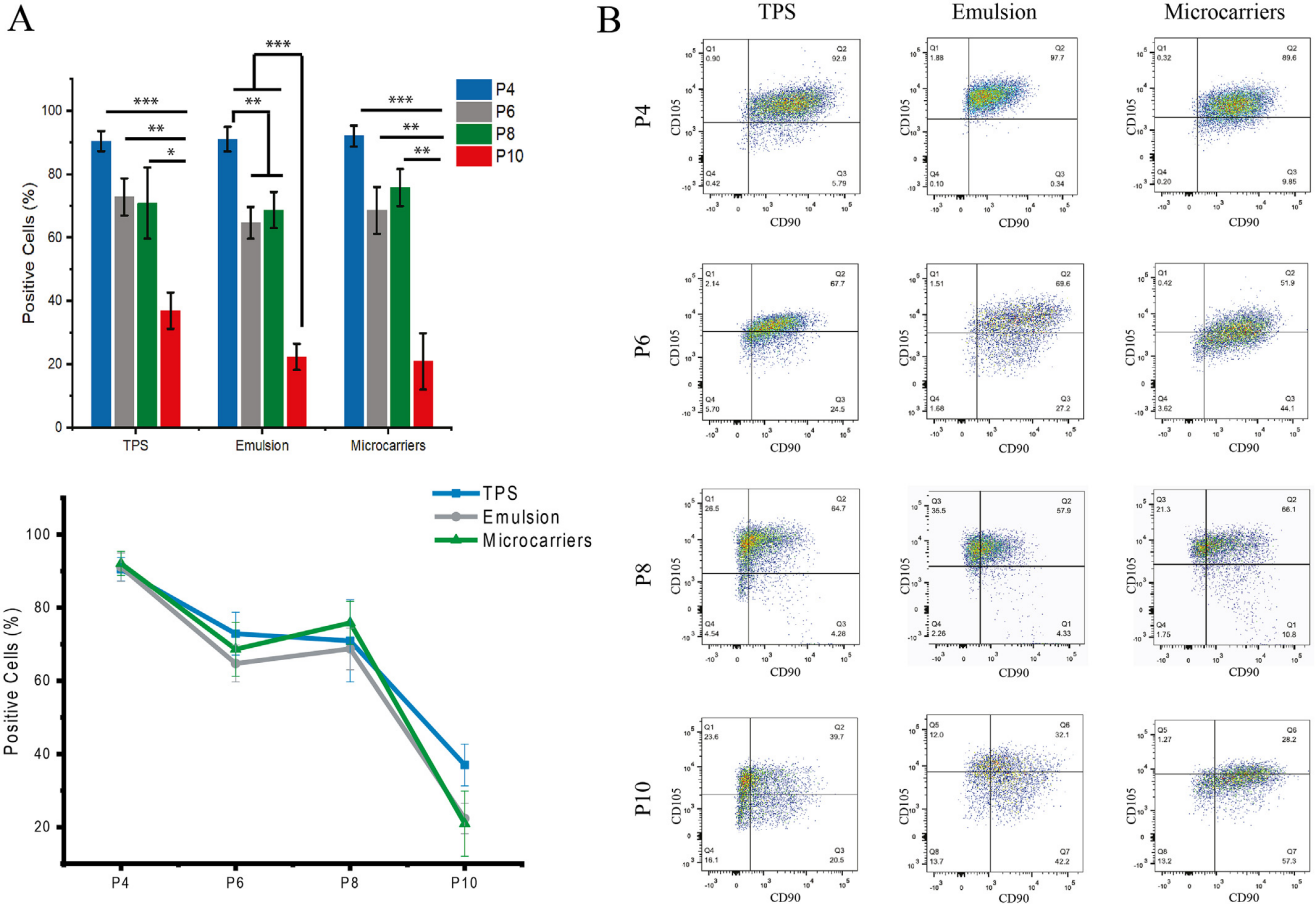
MSC expression of surface markers at different time points, when cultured on TPS, bioemulsions and solid microcarriers. A) (Top) Bar chart of the percentage of MSCs co-expressing CD73, CD90 and CD105, as a function of passage time and (Bottom) corresponding line chart. Error bars are S.E.; n ​≥ ​3. B) Representative examples of dot plots of CD105 against CD90 expression, with positive gating indicated.

The expression of typical MSC surface markers, including CD105, CD90 and CD73, was characterized next, via immuno-phenotyping flow cytometry. MSCs gradually lost the co-expression of these three markers at late passage times, with significantly reduced triple co-expression at P10 compared to P4 (Fig. 6). However, no significant difference was observed between substrates at any given time point. Analysis of each marker individually, indicated that high CD73 expression was maintained on all substrates throughout the culture time, whereas the expressions of both CD90 and CD105 was reduced at later passages (Supplementary Fig. S6). We noted some variation in the expression of these markers at P6 and P8, but below the threshold of statistical significance, followed by a substantial decrease in expression at P10. The downregulation of CD90 and CD105 has been proposed to be associated with differentiation [[66], [67], [68], [69], [70], [71]]. The downregulation of these markers is therefore not unexpected, and correlates with the change in MSC adhesion and morphology, as well as reduced proliferation, observed at later culture times. It is also worth noting that MSCs have been reported to display comparatively high autofluorescence, due to endogenous fluorophores such as nicotinamide adenine dinucleotide (NADH), flavin adenine dinucleotide (FAD) or structural proteins including COL and elastin [72,73]. Increased cell sizes at later passages could therefore increase autofluorescence, and may mask the signal of weakly expressed surface markers (as gated against FMO controls). As shown in Fig. 6B, the gating of MSCs that co-expressed CD90 and CD105 on bioemulsions and microcarriers was generally higher than that of TPS, therefore potentially contributing to the slight decrease in expression of these markers on these substrates. However, in the histogram of population for each individual marker remained comparable (Supplementary Fig. S6). Overall, MSCs on TPS displayed slightly reduced expression of CD90 and CD105 at passage 10 (compared to passage 4).

**Fig. 7:**
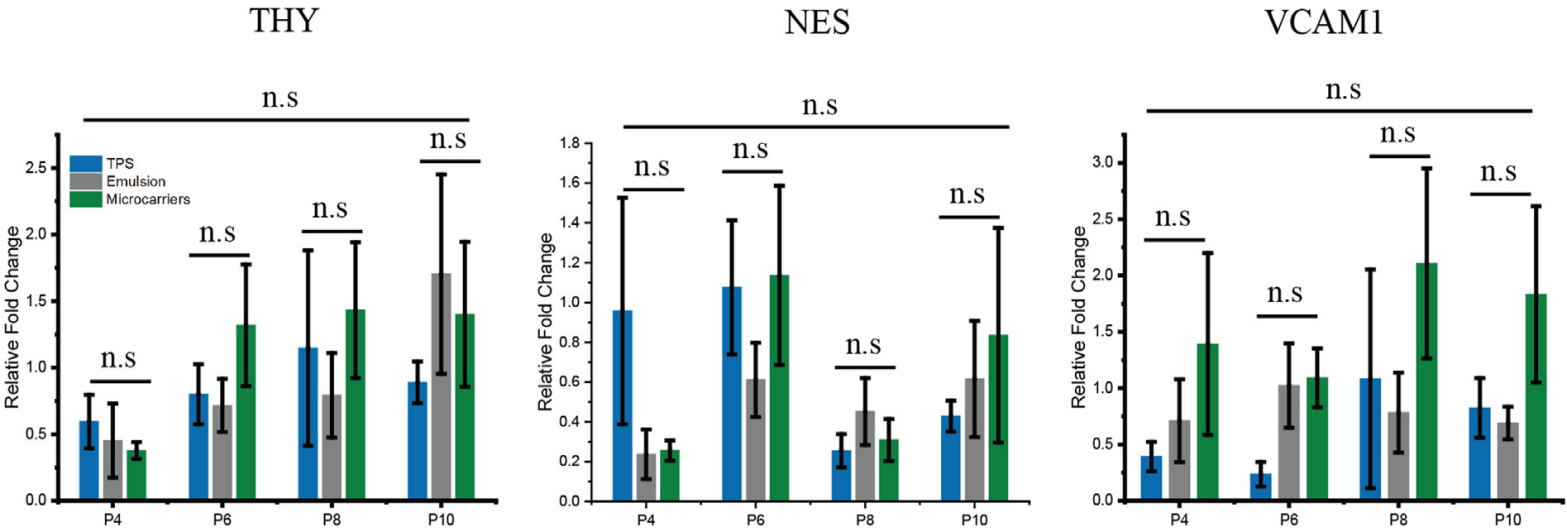
MSCs retain comparable levels of expression of stemness-associated genes on TPS, bioemulsions and solid microcarriers over long-term culture. RNA was directly extracted from MSCs cultured on TPS, bioemulsions and solid microcarriers at different passage times and reverse transcribed into cDNA for PCR experiments. Results are shown as relative fold change of gene expressed by MSCs on different substrates relative to that of newly thawed MSCs on TPS. Error bars are S.E.; n ​≥ ​3.

Considering the retention of stemness-associated surface markers observed until P10 for MSCs cultured on TPS, bioemulsions and solid microcarriers, the expression of stemness markers THY, NES and VCAM-1 was monitored next. At the gene expression level, few significant differences were observed between cells cultured on the different substrates throughout the culture time (Fig. 7). Interestingly, THY, also known as CD90, which had displayed downregulated surface marker expression at later passage in flow cytometry experiments, was found to be upregulated at the gene expression level by RT-PCR, suggesting that such high THY expression level does not translate into surface marker presentation, within the time frame studied. In turn, the neural marker Nestin, reported to be upregulated upon culture on soft substrates [74], remained expressed at low levels on all substrates at all passage times, including on bioemulsions. VCAM-1, also known as CD106, is an adhesion molecule that has been reported to mediate homing, migration and adhesion of MSCs [75,76]. The expression of this molecule was also found to be largely unaltered on the different substrates studied, at different passage times. Overall, our data demonstrate that, despite the macroscopically soft character of bioemulsions (and low viscosity of the oil selected for this study), the mechanical strength of the protein nanosheets self-assembled is sufficient to sustain cell adhesion and retain a stem cell phenotype, with little evidence for the loss of surface expression markers or changes in the expression of stemness-associated genes, beyond their variation as a function of passage time.

**Fig. 8:**
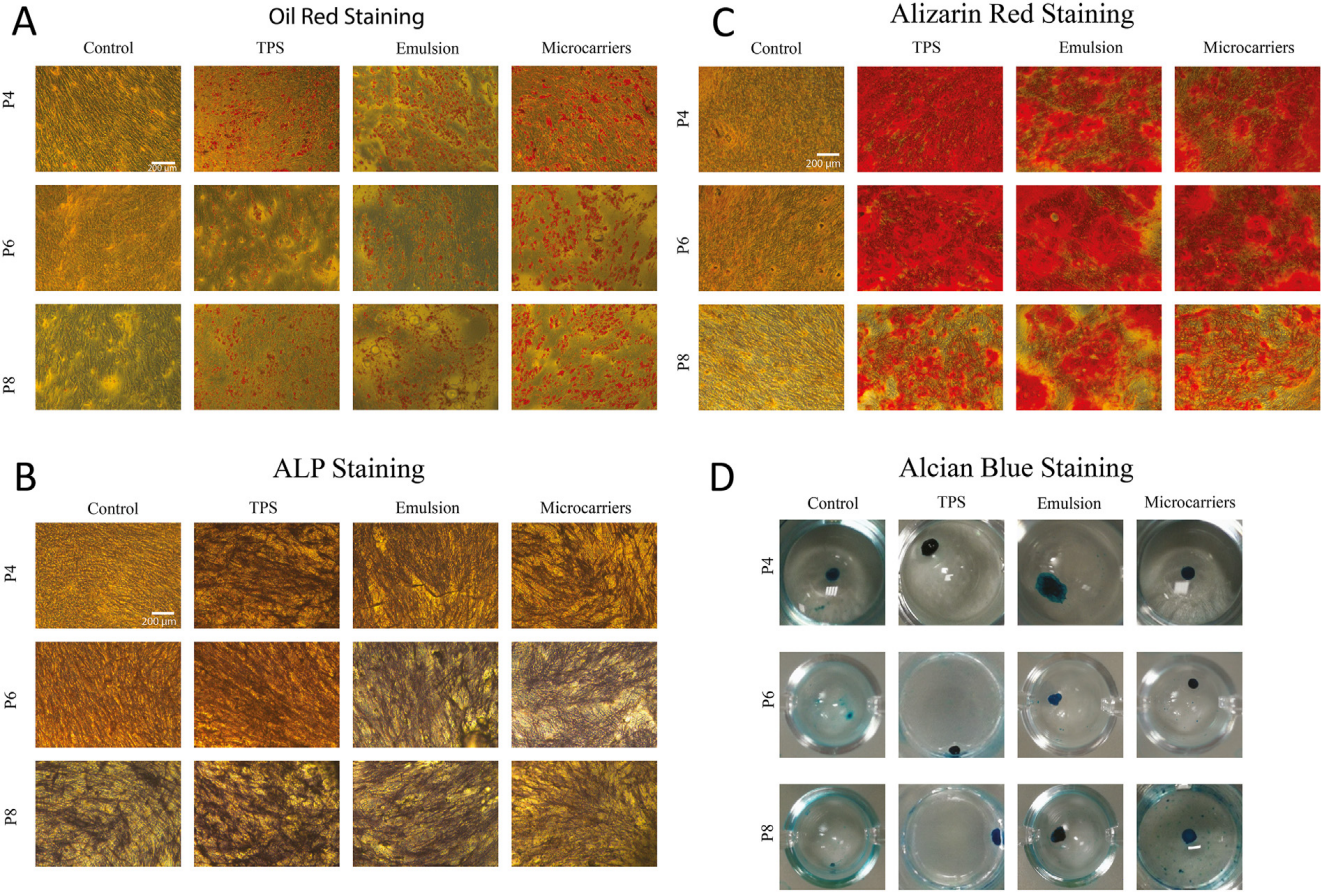
MSCs retain a multipotent phenotype after long-term culture on TPS, bioemulsions and solid microcarriers. MSCs were harvested from different substrates and subsequently cultured on TPS in differentiation media (14 days for adipogenic and osteogenic differentiation, 21 days for chondrogenesis). Bright field images of (A) Oil Red staining, (B) ALP staining, (C) Alizarin Red staining and (D) Alcian Blue stainings of corresponding samples. Controls were MSCs of the same passage harvested from TPS and cultured in growth medium (adipogenesis and osteogenesis) or DMEM (chondrogenesis).

To confirm the retention of multi-potency of MSCs cultured on bioemulsions, MSC differentiation towards three lineages (adipogenic, osteogenic and chondrogenic) was induced in corresponding differentiation media. As the ECM biochemistry and mechanics are also able to regulate differentiation [37,74], induction was triggered after transfer to TPS, from the corresponding long term expansion substrates. Adipogenesis (examined from oil red staining of lipid droplets, Fig. 8A) was clearly apparent with cells cultured on all three substrates, compared to the control group cultured in growth medium. Differentiation towards adipogenic lineages was also confirmed by RT-PCR, through the clear upregulation of the adipogenesis marker genes FABP4 and LPL. No statistically significant difference was found between expression of these genes in cells cultured on the different substrates studied at the same passage (Supplementary Fig. S7A). However, it was clear that MSCs gradually lost their adipogenic potential on prolonged culture. Since MSCs do not express FABP4 and LPL when culture in growth medium, the relative fold change of gene expression was normalised to P4 MSCs on TPS in differentiation medium. The expression of FABP4 on MSCs growing on microcarriers and bioemulsions was significantly downregulated at P8 compared to P4, however it was higher on TPS at P4. Although FABP4 is an early indicator of adipogenesis, this reduction does not necessarily imply that adipogenesis was halted as LPL expression at the same passages remained comparable, consistent with Oil Red stainings. Overall, such high expression levels compared to controls cultured in expansion medium indicate the retention of adipogenic potential even for MSCs cultured at P8 on the different substrates.

The induction of osteogenesis by MSCs has been identified as a multi-step process. It is initiated with a proliferation stage until the establishment of confluency, followed by a matrix maturation step, at which point the expression of ALP and RUNX2 becomes detectable [77,78]. The expression of RUNX2 indicates MSC commitment towards osteogenic lineages, resulting in the activation of the expression of type I COL and triggering matrix mineralisation, including OCN and osteopontin deposition, as well as calcium deposition [79,80]. Early osteogenesis was characterised by ALP staining. As shown in Fig. 8B, similar ALP activity can be observed for cells cultured on all three substrates, even at late passages, confirming their ability to differentiate into osteoblasts. Interestingly, ALP activity was also observed in the control group at passage 8 in growth medium, which is consistent with previous findings [74] showing that prolonged culture of MSCs on rigid substrates (TPS) induces osteogenic differentiation. Matrix mineralisation through calcium deposition was characterised via Alizarin Red staining (Fig. 8C). Although calcium deposition was observed on all substrates, a clear decrease occurs after prolonged culture (P8). However, trends in gene expression were less clear (Supplementary Fig. S7B), with no significant changes in the expression of RUNX2, BMP-2, COL1A1 and ALP, as a function of substrate type or passage number. BMP-2 has been widely studied and recognised as an important factor stimulating RUNX2 expression [80,81], which subsequently activates COL1A1. Therefore, the up-regulation of BMP2 expression and down-regulation of RUNX2 and COL1A1 expression at later passages may suggest that the osteogenesis process was slowing down at passage 8, although this was not significant. The role of BMP-2 in regulating matrix mineralisation has also been widely demonstrated [80]. COL1A1, ALP and RUNX2 expression was reported to decline upon initiation of matrix mineralisation [82]. Thus RT-PCR results may reflect a complex balance of differential regulation of matrix remodelling genes. In addition, although Alizarin Red staining indicated a reduction in mineralisation at P8, ALP activity was found to increase. These results are in agreement with the literature, with reports suggesting that cell senescence may promote the osteogenic differentiation [55,83,84], although others suggest the promotion of adipogenic commitment at later passages [59,60]. It is worth noting that in other reports, senescence impaired differentiation towards all lineages [73]. In our study, ALP activity at P8 was similar to P4 whereas the calcium deposition was reduced. Therefore, our results indicate that osteogenesis slowed down at P8 (Fig. 8B and C). However, overall the osteogenic potential of MSCs cultured on the three substrates studied was comparable.

Finally, chondrogenesis was induced in spheroid cultures, and Alcian Blue staining was performed to characterise the secretion of the cartilage matrix proteoglycan aggrecan. The formation of spheroids was observed in all conditions. Cells cultured in basal DMEM displayed lighter staining, whilst cells cultured in differentiation medium showed a dark blue staining indicative of abundant aggrecan deposition (Fig. 8D). The secretion of aggrecan seemed lighter at later passages, indicating a reduction in chondrogenic potential. Although no statistically significant differences in the expression of chondrogenesis-associated genes SOX9, COL2A1 and COL10A1 (between substrates and at P4 and P8), a gradual reduction in expression was observed at later passages (Supplementary Fig. S7C). COL2A1, in particular, which plays an important role in the regulation of the formation of articular cartilage was found to be more sensitive to passage numbers [85]. In comparison, SOX9 is expressed by proliferative chondrocytes in growth cartilage [86], and is reported to induce the secretion of the major cartilage matrix components type II and type X collagen, via the activation of the expression of COL2A1 and COL10A1 genes [79,85,86]. However, despite its role in promoting mineralisation of cartilage, COL10A1 is also associated with hypertrophic cartilage, which is undesirable for chondrogenesis at late stages [85,87]. Since SOX9 is hardly expressed by hypertrophic chondrocytes [86], the similar expression profile of SOX9 and COL10A1 in our experiments suggests that chondrocytes remained in a growth stage over the whole period of the experiments. Thus, the downregulation of COL10A1 in this case is proposed to indicate a loss of chondrogenic potential, to comparable level for the different substrates used in this study.

## 4 Conclusion

Therefore, our data demonstrate that bioemulsions stabilised by protein nanosheets such as PLL that display both strong interfacial mechanics and allow tethering or adsorption of ECM proteins, and potentially cell-adhesive ligands, enable the long term expansion of stem cells such as MSCs and the retention of their multi-potent phenotype. Our results demonstrate that the phenotype of MSCs cultured for up to the equivalent of six passages (47 days) on bioemulsions is comparable to that of MSCs cultured on solid microcarriers (and one of the current gold standards in the field, Synthemax® II). Our data also indicate that there is limited benefit of continuing to culture MSCs on 2D plastic as there is no systematic quantitative evidence for the retention of improved multipotency with this culture system and it is particularly restrictive in terms of scale up, automation and parallelisation. Indeed, although flow cytometry analysis indicates a slightly higher retention of triple surface marker co-expression for cells cultured on TPS (not statistically significant), this does not correlate with an increase in stemness gene expression (THY, NES, VCAM-1). The retention of multipotency is also comparable for MSCs cultured on bioemulsions and solid microcarriers compared to those cultured on TPS and the expression of some of the differentiation-associated genes is slightly upregulated on 3D microcarriers (in particular for adipo- and osteogenic differentiation). Cell densities are also increased on 3D microcarriers compared to TPS.

We however note that further medium optimisation (including for inducing differentiation) may highlight differences between the culture systems compared in our study. Similarly, the selection of microdroplets with homogenous and defined sizes might allow the identification of a more restricted set of conditions enabling a better (prolonged) control of MSC phenotype upon culture on bioemulsions. Other aspects requiring further examination include the stability of emulsions upon storage, transport and during long term expansion (although we note that the increase in droplet size observed in our system did not correlate with an altered phenotype, compared to cells cultured on homogenous microparticles), as well as optimisation of initial cell seeding and cell transfer between droplets.

Although the ability to culture and retain high proliferation levels at the surface of microdroplets may seem surprising, considering the importance of substrate mechanics on cell adhesion, spreading and phenotype, we note that the interfacial mechanical properties of protein nanosheets, in particular PLL nanosheets, are relatively stiff, at the local scale [47,48,88]. We propose that the dimensions of corresponding nanosheets (with thicknesses in the range of 10–20 ​nm, based on AFM data [48]) and their moduli (extrapolated to Young's moduli in the range of 1–100 ​MPa, based on interfacial rheology data [48,88]) are sufficient to resist forces generated by contractile and adhesive structures displaying comparable dimensions and stiffnesses (the acto-myosin networks and focal adhesion plaques; typically a few tens of nm across and with stiffnesses more difficult to directly quantify but unlikely to be in the GPa range).

These results also indicate that the use of mild enzymatic dissociation conditions for the resuspension of cells during passaging (such as Accutase) is not detrimental to MSC phenotype and that this process is mainly restrictive from a scale up and automation point of view rather than an intrinsic limiting parameter altering cell phenotype. These results contradict previous reports that proposed that enzymatic digestion is intrinsically harmful to cells and results in a loss of cell phenotypes [[89], [90], [91]]. Although this may be cell type dependent and enzymatic damage may be more evident with trypsin treatment, our data indicate that Accutase treatment is not significantly impairing the retention of MSC phenotype. However, enzymatic digestion and cell detachment, if not optimised, remains prone to error and increases risks of contaminations or cell/phenotype loss (i.e. if cells are not reseeded fast enough or if digestion is too long) and next generation cell manufacturing platforms should certainly focus on bypassing it. Overall, our results demonstrate that bioemulsions are a promising new generation of microcarriers for the long-term culture of adherent stem cells, including MSCs. In addition, bioemulsions offer significant advantages in terms of processing and scale up of cell culture and manufacturing, including significant cost reduction (the cost of the bioemulsions used in the present study was >10 fold lower than the cost of the microcarriers used). Indeed, cells can be recovered very readily via centrifugation (and potentially filtration or direct delivery of cell-laden oil droplets) and the oil phase contributes to the limitation of the use of microplastics in healthcare technologies, and could potentially be recycled after filtration. Finally, many oils, including fluorinated oils such as Novec 7500, but most importantly mineral and vegetal oils, remain considerably more affordable than currently used solid microcarriers (by a factor of at least 10 fold). Overall, bioemulsions constitute a unique opportunity to rethink the cell manufacturing pipeline and the processes associated with cell handling, recovery and post-culture processing for applications in tissue engineering, regenerative medicine, biotherapeutics production or synthetic meat production.

## Author statement

L.P. and J.E.G. developed the methodologies. J.E.G. developed the concept. L.P. carried out experiments and analysed the data gathered. J.E.G. supervised the project. L.P. and J.E.G. contributed to obtaining funding for this research. L.P. and J.E.G. wrote the manuscript and reviewed it. All authors have read and agreed to the published version of the article.

## Supplementary materials

Supplementary material associated with this article can be found, in the online version, at doi:.

## Data availability

Source datasets are available from the corresponding author on reasonable request and have been deposited in a QMUL repository.

## Declaration of competing interest

The authors declare the following financial interests/personal relationships which may be considered as potential competing interests. The authors wish to declare that they filed patent PCT/EP2018/097138, focusing on the broad use of liquid-liquid interfaces to culture adherent cells.
