# Supplementary material for: Long term expansion profile of mesenchymal stromal cells at protein nanosheet-stabilised bioemulsions for next generation cell culture microcarriers
Source: Mater Today Bio. 2021 Nov 16;12:100159. doi: 10.1016/j.mtbio.2021.100159 (PMC8605361; doi:10.1016/j.mtbio.2021.100159)
Supplement: Multimedia component 1 [file mmc1.pdf]

Long Term Expansion Profile of Mesenchymal Stromal Cells at Protein Nanosheet-Stabilised Bioemulsions for Next Generation Cell Culture Microcarriers

Supplementary Information

Lihui Peng[1,2] and Julien E. Gautrot[1,2*]

[1] Institute of Bioengineering and [2]School of Engineering and Materials Science, Queen Mary, University of London, Mile End Road, London, E1 4NS, UK.

[*]Corresponding author. j.gautrot@qmul.ac.uk

Supplementary Table S1. List of Primers for PCR

Gene Name	Assay ID
Beta-2-microglobulin (B2M)	Hs00187842_m1
Nestin	Hs04187831_g1
Thy-1 cell surface antigen (THY)	Hs00174816_m1
Vascular cell adhesion molecule 1 (VCAM1)	Hs01003372_m1
Fatty acid binding protein 4 (FABP4)	Hs01086177_m1
Lipoprotein lipase (LPL)	Hs00173425_m1
Runt related transcription factor 2 (RUNX2)	Hs01047973_m1
Collagen type I alpha 1 (COL1A1)	Hs00164004_m1
Bone morphogenetic protein 2 (BMP2)	Hs00154192_m1
Osteocalcin (OCN)	Hs01587814_g1
SRY-box 9 (SOX9)	Hs00165814_m1
Collagen type II alpha 1 chain (COL2A1)	Hs00264051_m1
Collagen type X alpha 1 chain (COL10A1)	Hs00166657_m1

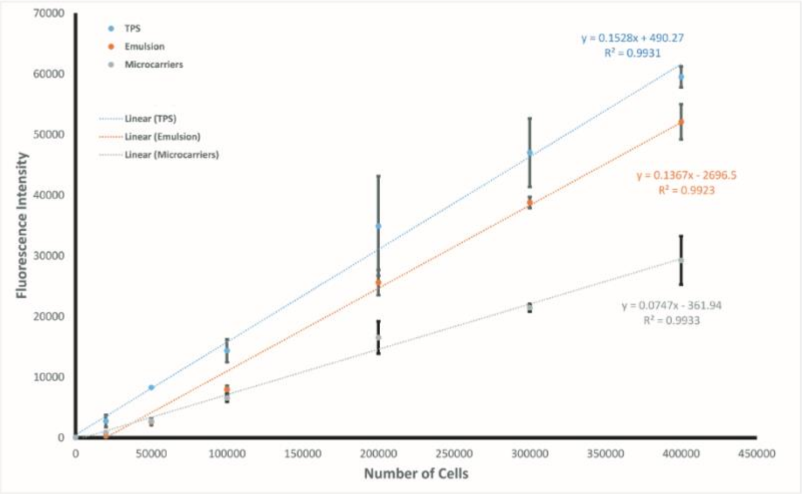

Supplementary Figure S1. Calibration curve for the conversion of CyQUANT™assay intensities in cell densities characterised in different culture systems. Linear regression fitting of the CyQUANT™ assay intensity data corresponding to MSCs introduced on TPS (blue), bioemulsion (orange) and solid microcarriers (grey). Error bars are s.e.m; n = 3.

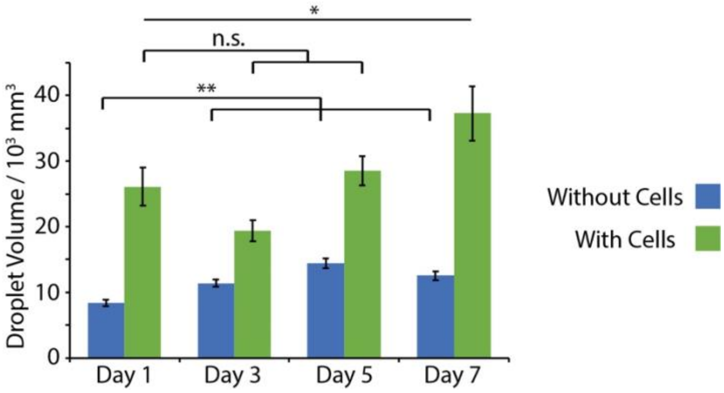

Supplementary Figure S2. Volume of PLL/FN stabilised microdroplets forming bioemulsions quantified over a period of 7 days. Error bars are s.e.m; n = 3.

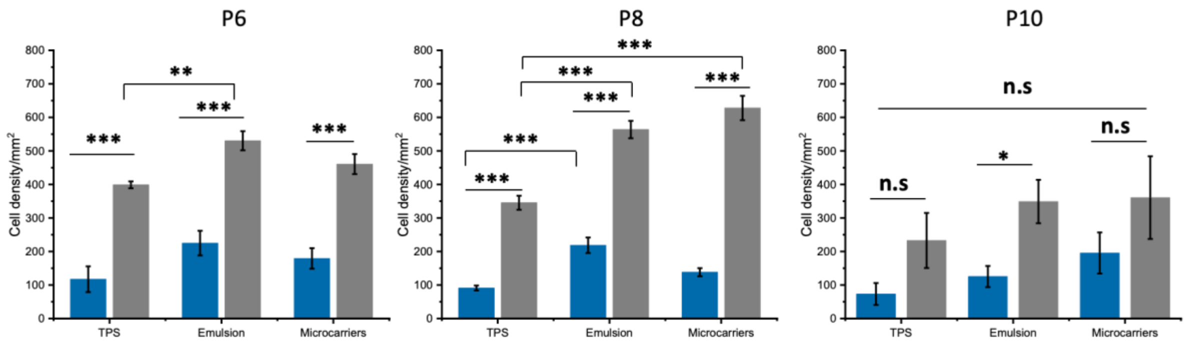

Supplementary Figure S3. MSC proliferation on TPS, bioemulsions and solid microcarriers at passage 6, 8 and 10 (corresponding to 6 subsequent passages, from MSCs cultured at P3). MSC densities were quantified via CyQUANT™ assay at days 1 (blue bars) and 5 (grey bars). Error bars are s.e.m; n represents number of experiments, n ≥ 3.

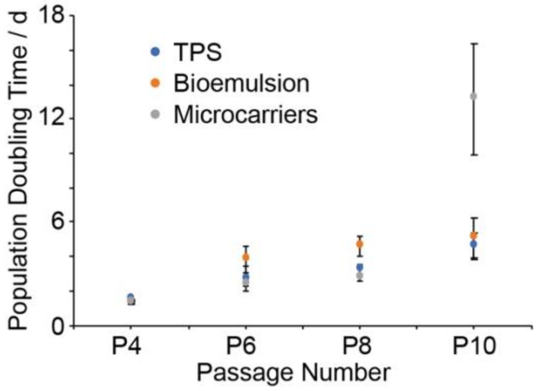

Supplementary Figure S4. MSCs population doubling times measured from CyQUANT™ assays, on different culture substrates, at different passage numbers. Error bars are s.e.m; n represents number of experiments, n ≥ 3.

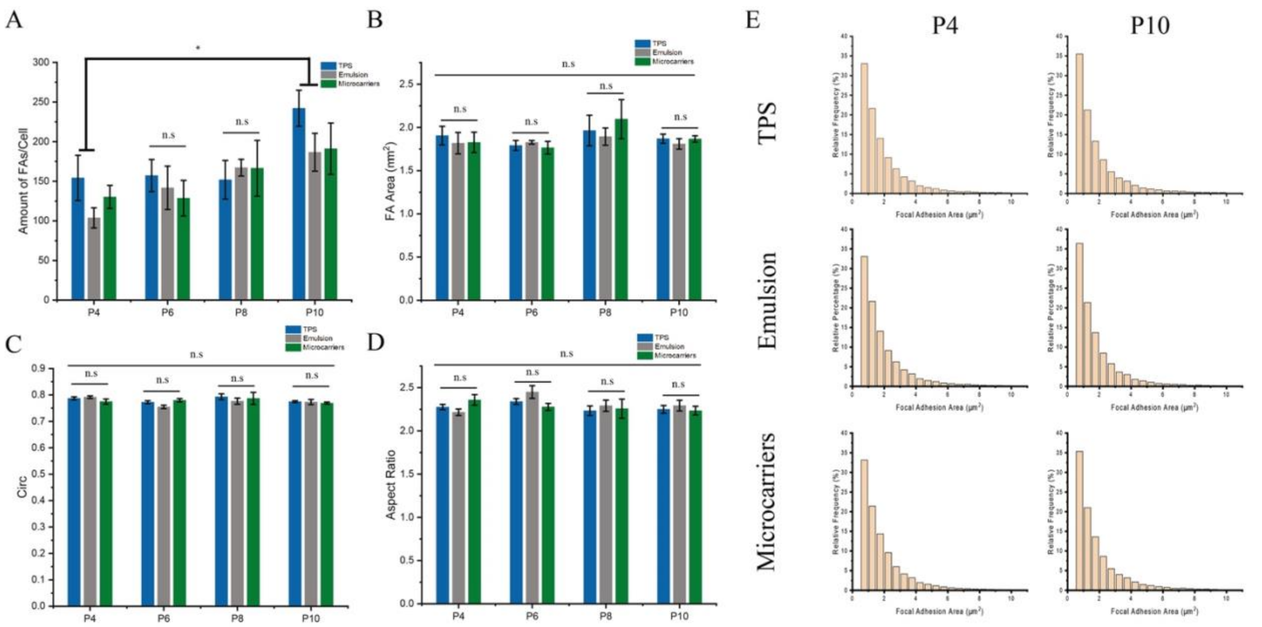

Supplementary Figure S5. MSC adhesions are comparable for cells cultured on TPS, bioemulsions and solid microcarriers over a broad period of cell expansion. Corresponding quantification of focal adhesion (from vinculin stainings) numbers (A), area (B), circularity (C) and aspect ratio (D). Error bars are s.e.m; n ≥ 3, more than 40 cells for each condition were measured in each experiment. E) Histogram of focal adhesion size distributions.

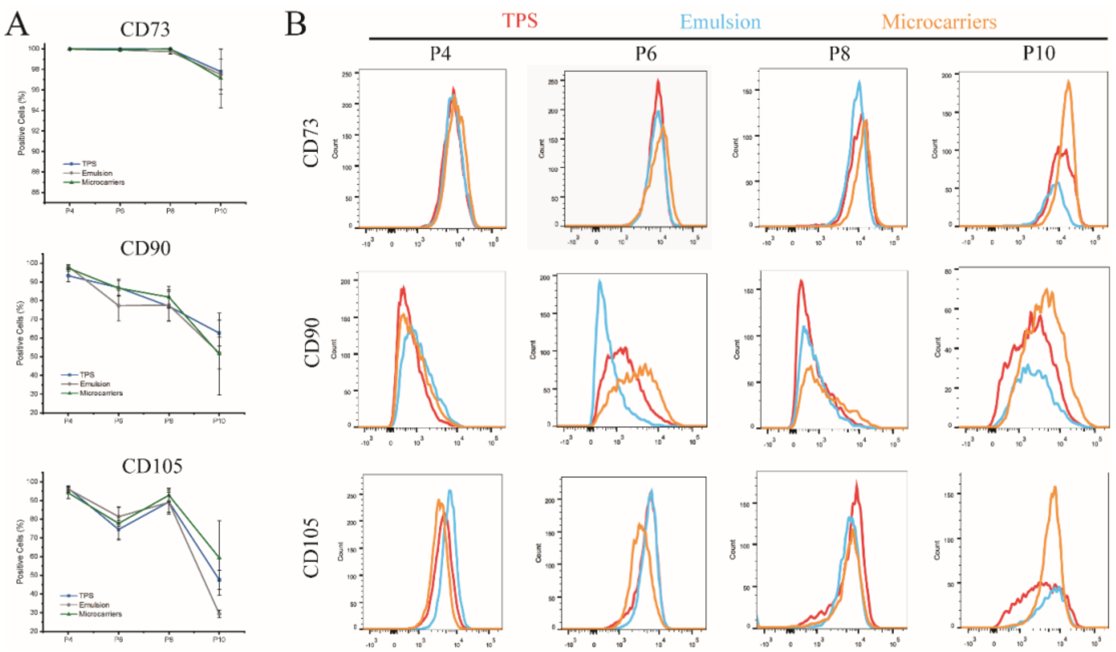

Supplementary Figure S6. MSCs retained comparable surface marker expression when cultured on TPS, bioemulsions and solid microcarriers for prolonged times. A) Evolution of the percentage of MSCs positive for individual markers (CD73, top; CD90, middle; CD105, bottom). Error bars are s.e.m; n ≥ 3. B) Representative examples of the fluorescence intensity distribution for individual surface markers for cells cultured on different substrates at different passage times. Red, TPS; blue, emulsion; orange, solid microcarriers.

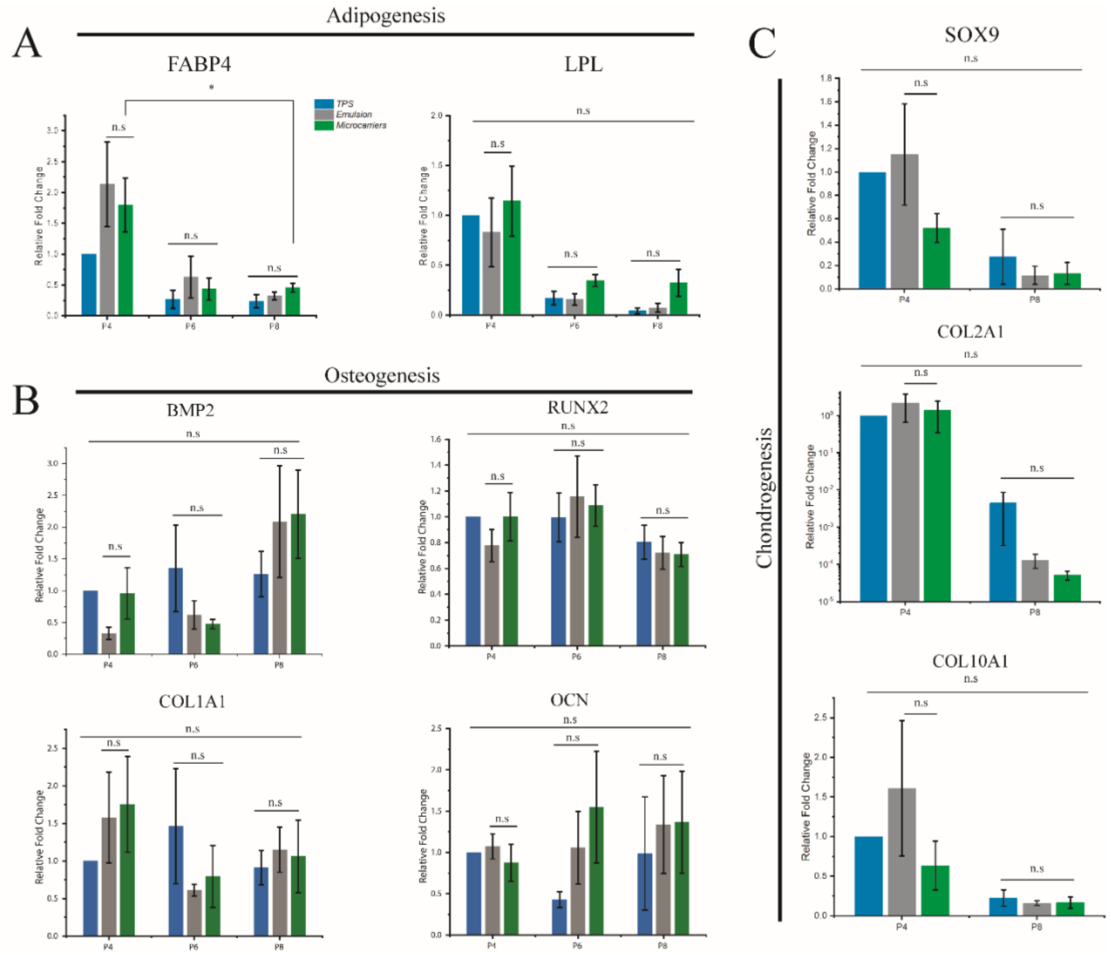

Supplementary Figure S7. Evaluation of MSC multipotency over long-term culture on TPS, bioemulsions and solid microcarriers, followed by differentiation in defined conditions and quantification of the expression of differentiation-associated genes by RT-PCR. MSCs were harvested from different substrates and subsequently cultured on TPS in differentiation media (14 days for adipogenic and osteogenic differentiation, 21 days for chondrogenesis). Relative fold changes of marker genes expressed by MSCs harvested from different substrates and subsequently differentiated on TPS, relative to that of P4 MSCs on TPS in differentiation medium. (A) adipogenesis; (B) osteogenesis and (C) chondrogenesis. Error bars are s.e.m; n ≥ 3.

Supplementary Table S2. Summary of statistical analysis for data presented in Figure 2B. Results of one-way and two way ANOVA are presented separately.

Without cells			
**One-way ANOVA**		**P-value**	**significance**
Day 1	Day 3	0.2505	n.s
Day 1	Day 5	0.03883	*
Day 3	Day 5	0.56449	n.s
Day 1	Day 7	0.19788	n.s
Day 3	Day 7	0.99784	n.s
Day 5	Day 7	0.66357	n.s

with cells			
**One-way ANOVA**		**P-value**	**significance**
Day 1	Day 3	0.82338	n.s
Day 1	Day 5	0.99848	n.s
Day 3	Day 5	0.74268	n.s
Day 1	Day 7	0.34997	n.s
Day 3	Day 7	0.11073	n.s
Day 5	Day 7	0.42135	n.s

Two-way ANOVA		p-value	significance
without cell	with cell	1.67E-06	***
Day 1	Day 3	0.99942	n.s
Day 1	Day 5	0.65275	n.s
Day 3	Day 5	0.58294	n.s
Day 1	Day 7	0.15916	n.s
Day 3	Day 7	0.12953	n.s
Day 5	Day 7	0.73396	n.s

Supplementary Table S3. Summary of statistical analysis for data presented in Figure 4.

**Figure 4B**					
**One-way ANOVA**		**Day1**		**Day 5**	
		**p-value**	**significance**	**p-value**	**significance**
bioemulsion	TPS	0.73433	n.s	0.01446	*
Microcarriers	TPS	0.25741	n.s	0.97525	n.s
Microcarriers	bioemulsion	0.0973	n.s	0.01148	*
		**TPS**	**bioemulsion**	**Microcarriers**	
**day 1**	**day 5**	7.01E-08	2.16E-06	1.21E-06	
		***	***	***	

**Figure 4C**					
**One-way ANOVA**		**Day1**		**Day 5**	
bioemulsion	TPS	0.19034	n.s	0.02568	*
Microcarriers	TPS	0.02159	*	0.002	**
Microcarriers	bioemulsion	0.38016	n.s	0.42667	n.s
		**TPS**	**bioemulsion**	**Microcarriers**	
**day 1**	**day 5**	7.01E-08	2.16E-06	1.21E-06	
		***	***	***	

Supplementary Table S4. Summary of statistical analysis for data presented in Figure 5B.

One-way ANOVA			
**P4**		**p-value**	**significance**
TPS	Emulsion	0.17143	n.s
TPS	Microcarriers	0.77595	n.s
Emulsion	Microcarriers	0.44887	n.s
**P6**			
TPS	Emulsion	0.51215	n.s
TPS	Microcarriers	0.64855	n.s
Emulsion	Microcarriers	0.97098	n.s
**P8**			
TPS	Emulsion	0.59172	n.s
TPS	Microcarriers	0.99343	n.s
Emulsion	Microcarriers	0.6855	n.s
**P10**			
TPS	Emulsion	0.42575	n.s
TPS	Microcarriers	0.28222	n.s
Emulsion	Microcarriers	0.87647	n.s
**TPS**			
P4	P6	0.67298	n.s
P4	P8	0.79029	n.s
P6	P8	0.99371	n.s
P4	P10	1.77E-05	***
P6	P10	1.58E-04	***
P8	P10	5.81E-05	***
**Emulsion**			
P4	P6	0.99999	n.s
P4	P8	0.97666	n.s
P6	P8	0.9763	n.s
P4	P10	0.00103	**
P6	P10	7.82E-03	**
P8	P10	4.80E-02	*
**Microcarriers**			
P4	P6	0.91888	n.s
P4	P8	0.53885	n.s
P6	P8	0.88293	n.s
P4	P10	0.01093	*
P6	P10	3.09E-02	*
P8	P10	9.77E-02	*

**Two-way ANOVA**		**p-value**	**significance**
emulsion	TPS	0.36577	n.s
microcarriers	TPS	0.06707	n.s
microcarriers	emulsion	0.5994	n.s
P4	P6	0.39983	n.s
P4	P8	0.02352	*
P6	P8	0.5517	n.s
P4	P10	4.17E-09	***
P6	P10	0	***
P8	P10	8.18E-08	***

Supplementary Table S5. Summary of statistical analysis for data presented in Figure 5C.

One-way			
**P4**		**p-value**	**significance**
TPS	Emulsion	0.96258	n.s
TPS	Microcarriers	0.99995	n.s
Emulsion	Microcarriers	0.95976	n.s
**P6**			
TPS	Emulsion	0.73257	n.s
TPS	Microcarriers	0.46796	n.s
Emulsion	Microcarriers	0.16982	n.s
**P8**			
TPS	Emulsion	0.80777	n.s
TPS	Microcarriers	0.66719	n.s
Emulsion	Microcarriers	0.33513	n.s
**P10**			
TPS	Emulsion	0.52235	n.s
TPS	Microcarriers	0.9301	n.s
Emulsion	Microcarriers	0.81871	n.s
**TPS**			
P4	P6	0.79828	n.s
P4	P8	0.8841	n.s
P6	P8	0.99321	n.s
P4	P10	9.49E-01	n.s
P6	P10	9.76E-01	n.s
P8	P10	9.98E-01	n.s
**Emulsion**			
P4	P6	0.99999	n.s
P4	P8	0.97666	n.s
P6	P8	0.9763	n.s
P4	P10	0.70361	n.s
P6	P10	7.54E-01	n.s
P8	P10	4.39E-01	n.s
**Microcarriers**			
P4	P6	0.47136	n.s
P4	P8	0.44721	n.s
P6	P8	0.99997	n.s
P4	P10	0.92358	n.s
P6	P10	8.90E-01	n.s
P8	P10	8.93E-01	n.s
**Two-way ANOVA**			
emulsion	TPS	0.9914	n.s
microcarriers	TPS	0.49203	n.s
microcarriers	emulsion	0.422	n.s
P4	P6	0.4669	n.s
P4	P8	0.71194	n.s
P6	P8	0.95823	n.s
P4	P10	5.30E-01	n.s.
P6	P10	0.99923	n.s
P8	P10	9.82E-01	n.s

Supplementary Table S6. Summary of statistical analysis for data presented in Figure 6.

**One-way ANOVA**			
**P4**		**p-value**	**significance**
emulsion	microcarriers	0.80127	n.s
TPS	microcarriers	0.92538	n.s
TPS	emulsion	0.52193	n.s
**P6**			
emulsion	microcarriers	0.91663	n.s
TPS	microcarriers	0.8778	n.s
TPS	emulsion	0.68816	n.s
**P8**			
emulsion	microcarriers	0.81157	n.s
TPS	microcarriers	0.90366	n.s
TPS	emulsion	0.97951	n.s
**P10**			
emulsion	microcarriers	0.98908	n.s
TPS	microcarriers	0.2401	n.s
TPS	emulsion	0.29605	n.s
**TPS**			
P4	P6	0.22839	n.s
P4	P8	0.27474	n.s
P6	P8	0.99686	n.s
P4	P10	2.04E-04	***
P6	P10	3.11E-03	**
P8	P10	1.87E-02	*
**emulsion**			
P4	P6	7.07E-04	***
P4	P8	0.00418	**
P6	P8	0.90701	n.s
P4	P10	2.26E-07	***
P6	P10	1.06E-04	***
P8	P10	8.53E-05	***
**microcarriers**			
P4	P6	1.69E-01	n.s
P4	P8	0.55285	n.s
P6	P8	0.89765	n.s
P4	P10	4.97E-04	***
P6	P10	3.75E-03	**
P8	P10	3.83E-03	**
**Two-way ANOVA**			
emulsion	microcarriers	0.91409	n.s
microcarriers	TPS	0.95134	n.s
TPS	emulsion	0.99194	n.s
P4	P6	6.17E-05	***
P4	P8	0.00218	**
P6	P8	0.95681	n.s
P4	P10	0.00E+00	***
P6	P10	0	***
P8	P10	1.50E-09	***

Supplementary Table S7. Summary of statistical analysis for data presented in Figure 7.

One-way ANOVA											
**THY**				**NES**				**VCAM**			
**TPS**		**p-value**	**significance**	**TPS**		**p-value**	**significance**	**TPS**		**p-value**	**significance**
P4	P6	0.97993	n.s	P4	P6	0.39904	n.s	P4	P6	0.99278	n.s
P4	P8	0.7636	n.s	P4	P8	0.18308	n.s	P4	P8	0.7233	n.s
P6	P8	0.91439	n.s	P6	P8	0.9009	n.s	P6	P8	0.50092	n.s
P4	P10	0.95219	n.s	P4	P10	0.21736	n.s	P4	P10	0.90989	n.s
P6	P10	0.99826	n.s	P6	P10	0.93856	n.s	P6	P10	0.7498	n.s
P8	P10	0.96714	n.s	P8	P10	0.99958	n.s	P8	P10	0.97801	n.s
**Emulsion**											
P4	P6	0.97889	n.s	P4	P6	0.62342	n.s	P4	P6	0.90531	n.s
P4	P8	0.96224	n.s	P4	P8	0.67377	n.s	P4	P8	0.99906	n.s
P6	P8	0.99943	n.s	P6	P8	1	n.s	P6	P8	0.96282	n.s
P4	P10	0.29017	n.s	P4	P10	0.70528	n.s	P4	P10	0.99998	n.s
P6	P10	0.43463	n.s	P6	P10	0.99995	n.s	P6	P10	0.9107	n.s
P8	P10	0.5491	n.s	P8	P10	0.99994	n.s	P8	P10	0.99838	n.s
**Microcarriers**				**Microcarriers**				**Microcarriers**			
P4	P6	0.44783	n.s	P4	P6	0.5953	n.s	P4	P6	0.98662	n.s
P4	P8	0.39722	n.s	P4	P8	1	n.s	P4	P8	0.89247	n.s
P6	P8	0.99762	n.s	P6	P8	0.59738	n.s	P6	P8	0.68403	n.s
P4	P10	0.4225	n.s	P4	P10	0.86392	n.s	P4	P10	0.96503	n.s
P6	P10	0.99911	n.s	P6	P10	0.97318	n.s	P6	P10	0.80894	n.s
P8	P10	0.99995	n.s	P8	P10	0.86532	n.s	P8	P10	0.99108	n.s
**P4**				**P4**				**P4**			
Emulsion	TPS	0.87233	n.s	Emulsion	TPS	0.83955	n.s	Emulsion	TPS	0.88972	n.s
Microcarriers	TPS	0.73332	n.s	Microcarriers	TPS	0.40617	n.s	Microcarriers	TPS	0.40772	n.s
Microcarriers	Emulsion	0.96301	n.s	Microcarriers	Emulsion	0.73193	n.s	Microcarriers	Emulsion	0.60315	n.s
**P6**				**P6**				**P6**			
Emulsion	TPS	0.98045	n.s	Emulsion	TPS	0.66048	n.s	Emulsion	TPS	0.14156	n.s
Microcarriers	TPS	0.49786	n.s	Microcarriers	TPS	0.99298	n.s	Microcarriers	TPS	0.1064	n.s
Microcarriers	Emulsion	0.39658	n.s	Microcarriers	Emulsion	0.59158	n.s	Microcarriers	Emulsion	0.98352	n.s
**P8**				**P8**				**P8**			
Emulsion	TPS	0.98045	n.s	Emulsion	TPS	0.51884	n.s	Emulsion	TPS	0.95946	n.s
Microcarriers	TPS	0.49786	n.s	Microcarriers	TPS	0.9494	n.s	Microcarriers	TPS	0.63697	n.s
Microcarriers	Emulsion	0.39658	n.s	Microcarriers	Emulsion	0.70098	n.s	Microcarriers	Emulsion	0.48745	n.s
**P10**				**P10**				**P10**			
Emulsion	TPS	0.60032	n.s	Emulsion	TPS	0.9368	n.s	Emulsion	TPS	0.98706	n.s
Microcarriers	TPS	0.82913	n.s	Microcarriers	TPS	0.73794	n.s	Microcarriers	TPS	0.46708	n.s
Microcarriers	Emulsion	0.92872	n.s	Microcarriers	Emulsion	0.91325	n.s	Microcarriers	Emulsion	0.38737	n.s

TWO-way ANOVA											
**THY**		**p-value**	**significance**	**NES**		**p-value**	**significance**	**VCAM**		**p-value**	**significance**
TPS	emulsion	0.947	n.s	TPS	emulsion	0.82167	n.s	TPS	emulsion	0.73959	n.s
TPS	microcarriers	0.61133	n.s	TPS	microcarriers	0.64961	n.s	TPS	microcarriers	0.0161	*
emulsion	microcarriers	0.79344	n.s	emulsion	microcarriers	0.95461	n.s	emulsion	microcarriers	0.08272	n.s
P6	P4	0.52311	n.s	P6	P4	0.50538	n.s	P6	P4	0.99965	n.s
P8	P4	0.28749	n.s	P8	P4	0.14356	n.s	P8	P4	0.61979	n.s
P8	P6	0.95306	n.s	P8	P6	0.78065	n.s	P8	P6	0.49383	n.s
P10	P4	0.07353	n.s	P10	P4	0.29372	n.s	P10	P4	0.79831	n.s
P10	P6	0.6069	n.s	P10	P6	0.95333	n.s	P10	P6	0.69292	n.s
P10	P8	0.90977	n.s	P10	P8	0.97948	n.s	P10	P8	0.98748	n.s

Supplementary Table S8. Summary of statistical analysis for data presented in Supplementary Figure S2.

One Way ANOVA				
	Without Cells		With Cells	
	Prob	Sig	Prob	Sig
Days 3/1	0.00144	**	0.23237	n.s.
Days 5/1	7.81E-12	***	0.90063	n.s.
Days 5/3	0.00242	**	0.04896	n.s.
Days 7/1	2.98E-06	***	0.02649	*
Days 7/3	0.5001	n.s.	4.15E-05	***
Days 7/5	0.14442	n.s.	0.1312	n.s.

Two Way ANOVA		
	Prob	Sig
Days 3/1	0.99534	n.s.
Days 5/1	0.00137	**
Days 5/3	6.33E-04	***
Days 7/1	0.00292	**
Days 7/3	0.00142	**
Days 7/5	0.99946	n.s.
With/Without Cells	2.22E-16	***

Supplementary Table S9. Summary of statistical analysis for data presented in Supplementary Figure S3.

**P6-Day 1**		**p-value**		**significance**
Emulsion	TPS	0.11406		n.s
Microcarriers	TPS	0.45138		n.s
Emulsion	Microcarriers	0.64265		n.s
**P6-Day 5**				
Emulsion	TPS	0.00481		**
Microcarriers	TPS	0.20964		n.s
Emulsion	Microcarriers	0.14533		n.s
**P6**		TPS	Emulsion	Microcarriers
day 1	day 5	3.44E-05	6.37E-05	6.40E-05
		***	***	***

**P8-Day 1**		**p-value**		**significance**
Emulsion	TPS	1.04E-04		***
Microcarriers	TPS	0.11698		n.s
Emulsion	Microcarriers	0.00649		**
**P8-Day 5**				
Emulsion	TPS	1.95E-04		***
Microcarriers	TPS	1.16E-05		***
Emulsion	Microcarriers	0.27716		n.s
**P8**		**TPS**	**Emulsion**	**Microcarriers**
day 1	day 5	6.51E-06	1.70E-06	1.58E-07
		***	***	***

**P10-Day 1**		**p-value**		**significance**
Emulsion	TPS	6.87E-01		n.s
Microcarriers	TPS	0.15556		n.s
Emulsion	Microcarriers	0.51252		n.s
**P10-Day 5**				
Emulsion	TPS	6.61E-01		n.s
Microcarriers	TPS	6.07E-01		n.s
Emulsion	Microcarriers	0.99571		n.s
**P10**		**TPS**	**Emulsion**	**Microcarriers**
day 1	day 5	1.01E-01	1.12E-02	2.58E-01
		n.s	**	n.s

Supplementary Table S10. Summary of statistical analysis for data presented in Supplementary Figure S5A.

**One-way ANOVA**			
**FA number**			
**TPS**		**p-value**	**significance**
P4	P6	0.74901	n.s
P4	P8	0.784	n.s
P6	P8	0.99846	n.s
P4	P10	0.01502	*
P6	P10	0.11037	n.s
P8	P10	0.05002	n.s
**Emulsion**			
P4	P6	0.09924	n.s
P4	P8	0.04552	*
P6	P8	0.99901	n.s
P4	P10	0.01064	*
P6	P10	0.78461	n.s
P8	P10	0.80873	n.s
**Microcarriers**			
P4	P6	0.99997	n.s
P4	P8	0.74405	n.s
P6	P8	0.75163	n.s
P4	P10	0.47754	n.s
P6	P10	0.4919	n.s
P8	P10	0.93066	n.s
**P4**			
Emulsion	TPS	0.77814	n.s
Microcarriers	TPS	0.91673	n.s
Microcarriers	Emulsion	0.50685	n.s
**P6**			
Emulsion	TPS	0.97276	n.s
Microcarriers	TPS	0.61773	n.s
Microcarriers	Emulsion	0.49021	n.s
**P8**			
Emulsion	TPS	0.88982	n.s
Microcarriers	TPS	0.90789	n.s
Microcarriers	Emulsion	0.99977	n.s
**P10**			
Emulsion	TPS	0.24101	n.s
Microcarriers	TPS	0.38726	n.s
Microcarriers	Emulsion	0.99192	n.s
**Two-way ANOVA**			
**FA number**		**p-value**	**significance**
Emulsion	TPS	0.60921	n.s
Microcarriers	TPS	0.46905	n.s
Microcarriers	Emulsion	0.96051	n.s
P4	P6	0.33823	n.s
P4	P8	0.06933	n.s
P6	P8	0.91725	n.s
P4	P10	5.49E-05	***
P6	P10	0.01717	*
P8	P10	0.0466	*

Supplementary Table S11. Summary of statistical analysis for data presented in Supplementary Figure S5B.

One-way ANOVA			
**FA area**			
**TPS**		**p-value**	**significance**
P4	P6	0.9757	n.s
P4	P8	0.94296	n.s
P6	P8	0.77819	n.s
P4	P10	1	n.s
P6	P10	0.97528	n.s
P8	P10	0.94373	n.s
**Emulsion**			
P4	P6	0.99988	n.s
P4	P8	0.92798	n.s
P6	P8	0.95814	n.s
P4	P10	0.99989	n.s
P6	P10	0.99914	n.s
P8	P10	0.90377	n.s
**Microcarriers**			
P4	P6	0.99148	n.s
P4	P8	0.57548	n.s
P6	P8	0.45597	n.s
P4	P10	0.9983	n.s
P6	P10	0.97592	n.s
P8	P10	0.77091	n.s
**P4**			
Emulsion	TPS	0.94887	n.s
Microcarriers	TPS	0.96571	n.s
Microcarriers	Emulsion	0.99819	n.s
**P6**			
Emulsion	TPS	0.89081	n.s
Microcarriers	TPS	0.9399	n.s
Microcarriers	Emulsion	0.71377	n.s
**P8**			
Emulsion	TPS	0.95043	n.s
Microcarriers	TPS	0.85247	n.s
Microcarriers	Emulsion	0.69036	n.s
**P10**			
Emulsion	TPS	0.67587	n.s
Microcarriers	TPS	0.99933	n.s
Microcarriers	Emulsion	0.76311	n.s
**Two-way ANOVA**			
**FA area**			
Emulsion	TPS	0.90181	n.s
Microcarriers	TPS	0.96496	n.s
Microcarriers	Emulsion	0.77701	n.s
P4	P6	0.98864	n.s
P4	P8	0.38676	n.s
P6	P8	0.25553	n.s
P4	P10	9.98E-01	n.s
P6	P10	0.96112	n.s
P8	P10	0.51872	n.s

Supplementary Table S12. Summary of statistical analysis for data presented in Supplementary Figure S5C.

**One-way ANOVA**			
**Circ**			
**TPS**		**p-value**	**significance**
P4	P6	0.67148	n.s
P4	P8	0.90563	n.s
P6	P8	0.30216	n.s
P4	P10	0.7373	n.s
P6	P10	0.99809	n.s
P8	P10	0.33446	n.s
**Emulsion**			
P4	P6	0.06539	n.s
P4	P8	0.60539	n.s
P6	P8	0.37887	n.s
P4	P10	0.48253	n.s
P6	P10	0.55063	n.s
P8	P10	0.99247	n.s
**Microcarriers**			
P4	P6	0.99578	n.s
P4	P8	0.92856	n.s
P6	P8	0.98515	n.s
P4	P10	0.99384	n.s
P6	P10	0.96999	n.s
P8	P10	0.86215	n.s
**P4**			
Emulsion	TPS	0.84487	n.s
Microcarriers	TPS	0.5283	n.s
Microcarriers	Emulsion	0.25843	n.s
**P6**			
Emulsion	TPS	0.36202	n.s
Microcarriers	TPS	0.64225	n.s
Microcarriers	Emulsion	0.1124	n.s
**P8**			
Emulsion	TPS	0.70397	n.s
Microcarriers	TPS	0.95612	n.s
Microcarriers	Emulsion	0.87937	n.s
**P10**			
Emulsion	TPS	0.98114	n.s
Microcarriers	TPS	0.83977	n.s
Microcarriers	Emulsion	0.91355	n.s
**Two-way ANOVA**			
**Circ**		p-value	significance
Emulsion	TPS	0.55026	n.s
Microcarriers	TPS	0.84318	n.s
Microcarriers	Emulsion	0.89283	n.s
P4	P6	0.33206	n.s
P4	P8	0.99954	n.s
P6	P8	0.24873	n.s
P4	P10	5.50E-01	n.s
P6	P10	0.97852	n.s
P8	P10	0.44852	n.s

Supplementary Table S13. Summary of statistical analysis for data presented in Supplementary Figure S5D.

One-way ANOVA			
**Aspect ratio**			
**TPS**		**p-value**	**significance**
P4	P6	0.72259	n.s
P4	P8	0.94167	n.s
P6	P8	0.3955	n.s
P4	P10	0.99164	n.s
P6	P10	0.56422	n.s
P8	P10	0.99319	n.s
**Emulsion**			
P4	P6	0.07652	n.s
P4	P8	0.78266	n.s
P6	P8	0.28557	n.s
P4	P10	0.79774	n.s
P6	P10	0.32099	n.s
P8	P10	1	n.s
**Microcarriers**			
P4	P6	0.88507	n.s
P4	P8	0.77288	n.s
P6	P8	0.99779	n.s
P4	P10	0.73708	n.s
P6	P10	0.98528	n.s
P8	P10	0.99726	n.s
**P4**			
Emulsion	TPS	0.70567	n.s
Microcarriers	TPS	0.36646	n.s
Microcarriers	Emulsion	0.10825	n.s
**P6**			
Emulsion	TPS	0.74306	n.s
Microcarriers	TPS	0.41626	n.s
Microcarriers	Emulsion	0.17803	n.s
**P8**			
Emulsion	TPS	0.8447	n.s
Microcarriers	TPS	0.97335	n.s
Microcarriers	Emulsion	0.94881	n.s
**P10**			
Emulsion	TPS	0.80738	n.s
Microcarriers	TPS	0.97926	n.s
Microcarriers	Emulsion	0.74576	n.s

Two-way ANOVA			
**Aspect ratio**		p-value	significance
Emulsion	TPS	0.66796	n.s
Microcarriers	TPS	0.89414	n.s
Microcarriers	Emulsion	0.92426	n.s
P4	P6	0.51433	n.s
P4	P8	0.97353	n.s
P6	P8	0.26073	n.s
P4	P10	9.82E-01	n.s
P6	P10	0.32519	n.s
P8	P10	0.99999	n.s

Supplementary Table S14. Summary of statistical analysis for data presented in Supplementary Figure S6.

One-way

**CD105**			
**p4**		p-value	significance
emulsion	TPS	0.9856	n.s
microcarriers	TPS	0.7020	n.s
microcarriers	emulsion	0.7659	n.s
**P6**			
emulsion	TPS	0.8262	n.s
microcarriers	TPS	0.9502	n.s
microcarriers	emulsion	0.9366	n.s
**P8**			
emulsion	TPS	0.9996	n.s
microcarriers	TPS	0.8857	n.s
microcarriers	emulsion	0.8738	n.s
**P10**			
emulsion	TPS	0.3777	n.s
microcarriers	TPS	0.6363	n.s
microcarriers	emulsion	0.9029	n.s

**CD73**			
**p4**		p-value	significance
emulsion	TPS	0.59513	n.s
microcarrier	TPS	1	n.s
microcarrier	emulsion	0.64284	n.s
**P6**			
emulsion	TPS	0.72141	n.s
microcarrier	TPS	0.67739	n.s
microcarrier	emulsion	1	n.s
**P8**			
emulsion	TPS	0.56831	n.s
microcarrier	TPS	0.98738	n.s
microcarrier	emulsion	0.48755	n.s
**P10**			
emulsion	TPS	0.99586	n.s
microcarrier	TPS	0.97588	n.s
microcarrier	emulsion	0.99325	n.s

**CD90**			
**p4**		p-value	significance
emulsion	TPS	0.22612	n.s
microcarriers	TPS	0.43398	n.s
microcarriers	emulsion	0.95328	n.s
**P6**			
emulsion	TPS	0.47577	n.s
microcarriers	TPS	0.99933	n.s
microcarriers	emulsion	0.43299	n.s
**P8**			
emulsion	TPS	0.99861	n.s
microcarriers	TPS	0.88955	n.s
microcarriers	emulsion	0.91073	n.s
**P10**			
emulsion	TPS	0.80717	n.s
microcarriers	TPS	0.78582	n.s
microcarriers	emulsion	0.99931	n.s

One-way ANOVA

TPS

**CD105**		p-value	significance	**CD73**		p-value	significance	**CD90**		p-value	significance
P4	P6	0.02938	*	P4	P6	1	n.s	P4	P6	0.87814	n.s
P4	P8	0.74898	n.s	P4	P8	1	n.s	P4	P8	0.33202	n.s
P6	P8	0.23225	n.s	P6	P8	1	n.s	P6	P8	0.70479	n.s
P4	P10	2.94E-05	***	P4	P10	6.49E-01	n.s	P4	P10	1.18E-02	*
P6	P10	0.00557	**	P6	P10	0.64929	n.s	P6	P10	0.04638	*
P8	P10	3.12E-04	***	P8	P10	7.14E-01	n.s	P8	P10	3.98E-01	n.s

One-way ANOVA

Emulsion

**CD105**		p-value	significance	**CD73**		p-value	significance	**CD90**		p-value	significance
P4	P6	0.04413	*	P4	P6	0.9998	n.s	P4	P6	0.09992	n.s
P4	P8	0.54644	n.s	P4	P8	0.99209	n.s	P4	P8	0.14537	n.s
P6	P8	0.52027	n.s	P6	P8	0.9972	n.s	P6	P8	0.99999	n.s
P4	P10	1.61E-07	***	P4	P10	4.96E-02	*	P4	P10	1.35E-03	**
P6	P10	5.90E-06	***	P6	P10	0.06935	n.s	P6	P10	0.0785	n.s
P8	P10	2.90E-06	***	P8	P10	1.25E-01	n.s	P8	P10	1.01E-01	n.s

One-way ANOVA

Microcarriers

**CD105**		p-value	significance	**CD73**		p-value	significance	**CD90**		p-value	significance
P4	P6	0.664	n.s	P4	P6	0.99989	n.s	P4	P6	0.8304	n.s
P4	P8	0.99981	n.s	P4	P8	1	n.s	P4	P8	0.71642	n.s
P6	P8	0.71598	n.s	P6	P8	0.99989	n.s	P6	P8	0.98014	n.s
P4	P10	0.01923	*	P4	P10	0.37706	n.s	P4	P10	0.03845	*
P6	P10	0.0527	n.s	P6	P10	0.29226	n.s	P6	P10	0.06973	n.s
P8	P10	0.02194	*	P8	P10	0.37706	n.s	P8	P10	0.21181	n.s

Two-way ANOVA

**CD105**		p-value	significance
Emulsion	TPS	0.79158	n.s
Microcarriers	TPS	0.96218	n.s
Microcarriers	Emulsion	0.92264	n.s
P4	P6	0.00623	**
P4	P8	0.78412	n.s
P6	P8	0.12802	n.s
P4	P10	1.92E-07	***
P6	P10	1.44E-08	***
P8	P10	0	***

**CD73**		p-value	significance
Emulsion	TPS	0.98776	n.s
Microcarriers	TPS	0.99309	n.s
Microcarriers	Emulsion	0.99925	n.s
P4	P6	0.99987	n.s
P4	P8	0.99969	n.s
P6	P8	0.99999	n.s
P4	P10	2.45E-02	*
P6	P10	2.23E-02	*
P8	P10	0.05132	n.s

**CD90**		p-value	significance
Emulsion	TPS	0.99902	n.s
Microcarriers	TPS	0.93312	n.s
Microcarriers	Emulsion	0.94923	n.s
P4	P6	0.11502	n.s
P4	P8	0.02721	*
P6	P8	0.8	n.s
P4	P10	9.01E-08	***
P6	P10	6.68E-05	***
P8	P10	0.00435	**

Supplementary Table S15. Summary of statistical analysis for data presented in Supplementary Figure S7A.

**One-way ANOVA**			
**FABP4**			
**TPS**		p-value	significance
P6	P8	0.85436	n.s
**Emulsion**		p-value	significance
P4	P6	0.16792	n.s
P4	P8	0.09491	n.s
P6	P8	0.92052	n.s
**Microcarriers**		p-	significance
P4	P6	0.0521	n.s
P4	P8	0.04887	*
P6	P8	0.99894	n.s
P4		p-value	significance
microcarriers	Emulsion	0.69425	n.s
P6		p-value	significance
emulsion	TPS	0.56412	n.s
Microcarriers	TPS	0.87611	n.s
Microcarriers	Emulsion	0.83764	n.s
P8		p-value	significance
emulsion	TPS	0.22848	n.s
Microcarriers	TPS	0.747	n.s
Microcarriers	Emulsion	0.53788	n.s
**Two-way ANOVA**		**p-value**	
Emulsion	TPS	0.04468	*
TPS	Microcarri	0.10731	n.s
Emulsion	Microcarri	0.86682	n.s
P6	P8	0.93928	n.s
P6	P4	3.51E-04	***
P8	P4	1.62E-04	***

**One-way ANOVA**			
**LPL**			
**TPS**		p-value	significance
P6	P8	0.18638	n.s
**Emulsion**		p-value	significance
P4	P6	0.12625	n.s
P4	P8	0.11011	n.s
P6	P8	0.96378	n.s
**Microcarriers**		p-value	significance
P4	P6	0.08522	n.s
P4	P8	0.10126	n.s
P6	P8	0.99751	n.s
P4		p-value	significance
microcarriers	Emulsion	0.54567	n.s
P6		p-value	significance
emulsion	TPS	0.98564	n.s
Microcarriers	TPS	0.16039	n.s
Microcarriers	Emulsion	0.12593	n.s
P8		p-value	significance
emulsion	TPS	0.97075	n.s
Microcarriers	TPS	0.11984	n.s
Microcarriers	Emulsion	0.16124	n.s
**Two-way ANOVA**		**p-value**	
Emulsion	TPS	0.3142	n.s
TPS	Microcarr	0.01959	*
Emulsion	Microcarr	0.25349	n.s
P6	P8	0.87825	n.s
P6	P4	3.35E-04	***
P8	P4	2.22E-04	***

Supplementary Table S16. Summary of statistical analysis for data presented in Supplementary Figure S7B.

One-way ANOVA

BMP2

**TPS**		p-value	significance
P6	P8	0.44717	n.s

**Emulsion**		p-value	significance
P4	P6	0.93962	n.s
P4	P8	0.08311	n.s
P6	P8	0.2621	n.s

**Microcarriers**		p-value	significan
P4	P6	0.8264	n.s
P4	P8	0.21989	n.s
P6	P8	0.1455	n.s

P4		p-value	significance
microcarriers	Emulsion	0.16155	n.s

P6		p-value	significance
emulsion	TPS	0.57149	n.s
Microcarriers	TPS	0.46172	n.s
Microcarriers	Emulsion	0.98015	n.s

P8		p-value	significance
emulsion	TPS	0.93436	n.s
Microcarriers	TPS	0.96137	n.s
Microcarriers	Emulsion	0.99561	n.s

**Two-way ANOVA**		**p-value**	
Emulsion	TPS	0.28245	n.s
TPS	Microcarriers	0.52784	n.s
Emulsion	Microcarriers	0.85238	n.s
P6	P8	0.05056	n.s
P6	P4	9.23E-01	n.s
P8	P4	1.42E-02	*

One-way

RUNX2

**TPS**		p-value	significance
P6	P8	0.44029	n.s

**Emulsion**		p-value	significance
P4	P6	0.38852	n.s
P4	P8	0.97765	n.s
P6	P8	0.32404	n.s

**Microcarriers**		p-value	significanc
P4	P6	0.9194	n.s
P4	P8	0.42548	n.s
P6	P8	0.25468	n.s

P4		p-value	significance
microcarriers	Emulsion	0.34046	n.s

P6		p-value	significance
emulsion	TPS	0.8686	n.s
Microcarriers	TPS	0.95121	n.s
Microcarriers	Emulsion	0.97361	n.s

P8		p-value	significance
emulsion	TPS	0.87293	n.s
Microcarriers	TPS	0.83492	n.s
Microcarriers	Emulsion	0.99672	n.s

**Two-way**		**p-value**	
Emulsion	TPS	0.97961	n.s
TPS	Microcarriers	0.97007	n.s
Emulsion	Microcarriers	0.88189	n.s
P6	P8	0.06533	n.s
P6	P4	4.48E-01	n.s
P8	P4	6.25E-01	n.s

One-way ANOVA

COL1A1

**TPS**		p-value	significance
P6	P8	0.50844	n.s

**Emulsion**		p-value	significance
P4	P6	0.28093	n.s
P4	P8	0.76356	n.s
P6	P8	0.67766	n.s

**Microcarriers**		p-value	significance
P4	P6	0.43297	n.s
P4	P8	0.63625	n.s
P6	P8	0.93938	n.s

P4		p-value	significance
microcarriers	Emulsion	0.84449	n.s

P6		p-value	significance
emulsion	TPS	0.47655	n.s
Microcarriers	TPS	0.62498	n.s
Microcarriers	Emulsion	0.96479	n.s

P8		p-value	significance
emulsion	TPS	0.88299	n.s
Microcarriers	TPS	0.95326	n.s
Microcarriers	Emulsion	0.98185	n.s

Two-way ANOVA		p-value	
Emulsion	TPS	0.99995	n.s
TPS	Microcarriers	0.96398	n.s
Emulsion	Microcarriers	0.95196	n.s
P6	P8	0.97671	n.s
P6	P4	9.77E-02	n.s
P8	P4	1.40E-01	n.s

One-way ANOVA

OCN

**TPS**		p-value	significance
P6	P8	0.32232	n.s

**Emulsion**		p-value	significance
P4	P6	0.99959	n.s
P4	P8	0.90378	n.s
P6	P8	0.8921	n.s

**Microcarriers**		p-value	significance
P4	P6	0.61884	n.s
P4	P8	0.82157	n.s
P6	P8	0.97246	n.s

P4		p-value	significance
microcarriers	Emulsion	0.48542	n.s

P6		p-value	significance
emulsion	TPS	0.62279	n.s
Microcarriers	TPS	0.24878	n.s
Microcarriers	Emulsion	0.74316	n.s

P8		p-value	significance
emulsion	TPS	0.92011	n.s
Microcarriers	TPS	0.92507	n.s
Microcarriers	Emulsion	0.99945	n.s

Two-way ANOVA		p-value	
Emulsion	TPS	0.43334	n.s
TPS	Microcarriers	0.33637	n.s
Emulsion	Microcarriers	0.96465	n.s
P6	P8	0.80304	n.s
P6	P4	9.95E-01	n.s
P8	P4	7.84E-01	n.s

Supplementary Table S17. Summary of statistical analysis for data presented in Supplementary Figure S7C.

One-way			
**SOX9**			
**Emulsio**		p-value	significance
P4	P8	0.12528	n.s
**Microcarriers**		p-value	significance
P4	P6	0.06613	n.s
**P4**		p-value	significance
microcar	Emulsion	0.25094	n.s
**P8**		p-value	significance
emulsion	TPS	0.79072	n.s
Microcar	TPS	0.75218	n.s
Microcar	Emulsion	0.99725	n.s
**Two-way ANOVA**		p-value	
TPS	Microcar	0.97946	n.s
Emulsion	TPS	0.45244	n.s
Emulsion	Microcar	0.38825	n.s
P8	P4	2.37E-02	*

One-way			
**COL2A1**			
**Emulsion**		p-value	significance
P4	P6	0.32709	n.s
**Microcarriers**		p-value	significance
P4	P6	0.31348	n.s
**P4**		p-value	significance
microcarrier	Emulsion	0.69482	n.s
**P8**		p-value	significance
emulsion	TPS	0.45523	n.s
Microcarrier	TPS	0.44355	n.s
Microcarrier	Emulsion	0.99968	n.s
**Two-way ANOVA**		p-value	
Emulsion	TPS	0.60991	n.s
TPS	Microcar	0.85571	n.s
Emulsion	Microcar	0.85034	n.s
P8	P4	8.42E-02	n.s

One-way			
**COL10A1**			
**Emulsion**		p-value	significance
P4	P6	0.25061	n.s
**Microcarriers**		p-value	significance
P4	P6	0.26102	n.s
**P4**		p-value	significance
microcarr	Emulsion	0.3661	n.s
**P8**		p-value	significance
emulsion	TPS	0.84121	n.s
Microcarr	TPS	0.80925	n.s
Microcarr	Emulsion	0.99784	n.s
**Two-way ANOVA**		p-value	
TPS	Microcarrie	0.95874	n.s
Emulsion	TPS	0.50956	n.s
Emulsion	Microcarrie	0.5209	n.s
P8	P4	7.45E-02	n.s
